# A Unified Histopathological Framework of Liver Fibrogenesis in Chronic Viral Hepatitis B, C and Coinfection

**DOI:** 10.3390/diseases14050165

**Published:** 2026-05-08

**Authors:** Alina Dumitrache (Păunescu), Nicoleta Anca Ionescu (Șuțan), Liliana Cristina Soare, Maria Cristina Ponepal, Ana Cătălina Țânțu, Monica Marilena Țânțu, Ileana Monica Baniță, Cătălina Gabriela Pisoschi

**Affiliations:** 1 Doctoral School, University of Medicine and Pharmacy of Craiova, Petru-Rareș Street No. 2, 200349 Craiova, Romania; alina.paunescu@upb.ro (A.D.); catalina.tantu8@gmail.com (A.C.Ț.); monica.banita@yahoo.com (I.M.B.); c_pisoschi@yahoo.com (C.G.P.); 2 Department of Natural Sciences, National University of Science and Technology Politehnica Bucharest, Piteşti University Centre, 1st Targul din Vale Str., 110040 Pitesti, Romania; liliana.soare@upb.ro (L.C.S.); maria.ponepal@upb.ro (M.C.P.); 3 Department of Medical Assistance and Physical Therapy, National University of Science and Technology Politehnica Bucharest, Piteşti University Centre, 1st Targul din Vale Str., 110040 Pitesti, Romania; marilena.tantu@upb.ro

**Keywords:** chronic hepatitis B, chronic hepatitis C, coinfection B+C, histopathologic, immunohistochemical

## Abstract

Background: Chronic hepatitis B and C remain major causes of progressive liver disease, while HBV–HCV coinfection is associated with accelerated fibrosis and hepatocellular injury. Methods: This study evaluated integrated biochemical, histopathological, and immunohistochemical features in patients with chronic hepatitis B (CHB, *n* = 29), chronic hepatitis C (CHC, *n* = 15), and CHB+C coinfection (CHB+C, *n* = 10). Liver biopsies were assessed using Ishak and METAVIR scoring systems, alongside immunohistochemical analysis of α-smooth muscle actin (α-SMA), transforming growth factor-β1 (TGF-β1), CD5L, and glial fibrillary acidic protein (GFAP), quantified by H-score. These findings were correlated with biochemical, hematological, and prognostic parameters. Results: Coinfected patients exhibited significantly higher serum ALT, AST, and GGT levels (*p* ≤ 0.011) and increased CD5L expression (median H-score 197.5 vs. 135 in CHB, *p* = 0.009), indicating enhanced macrophage-associated inflammatory activity. Although fibrosis stages were comparable across groups, median H-scores for α-SMA, TGF-β1, and GFAP showed a consistent upward trend in CHB+C, suggesting intensified profibrogenic signaling. Principal Component Analysis identified distinct biochemical clusters related to hepatocellular injury, hepatic functional impairment (synthetic and excretory axis), and lipid metabolism. Conclusions: These findings highlight a multidimensional pattern of liver injury in chronic viral hepatitis, with CHB+C coinfection amplifying profibrogenic and hepatocellular markers, both biochemically and histologically.

## 1. Introduction

Chronic viral hepatitis is a major public health problem worldwide with a significant impact on liver morbidity and mortality. For this reason, the World Health Organization aims to eradicate it by 2030 [1] through national and regional strategic plans [2].

Hepatitis B and C viruses (HBV, HCV) are responsible for most chronic hepatitis cases [3], and coinfection with both viruses (CHB+C) can significantly complicate the course of the disease, leading to faster progression to cirrhosis and hepatocellular carcinoma (HCC) with an increased mortality rate [2]. Chronic hepatitis B (CHB) and C (CHC) infections provoke ongoing liver injury that, through repeated cycles of inflammation and repair, leads to extracellular matrix accumulation, fibrosis, cirrhosis, and ultimately HCC [4]. Histopathological chronic viral hepatitis is characterized by interface hepatitis, portal inflammation, and fibrotic remodeling, features that reflect underlying cellular interactions between hepatocytes, immune cells, and hepatic stellate cells (HSCs) [5,6]. Patients with CHB may have a mortality rate four times higher than the general population, even though anti-CHB therapies have been improved [7].

The diagnosis and monitoring of chronic hepatitis are based on clinical, serological and biochemical evaluation, but the precise determination of the degree of liver damage requires histopathological and immunohistochemical investigations. Histopathological analysis provides essential information about inflammation, necrosis and fibrosis [8], while immunohistochemical evaluations allow the identification of the expression of specific markers of local immune cell activation [9]. In addition, liver biochemical tests provide data on liver function that correlates with the expression of the severity of liver damage [10].

HSCs are the principal source of myofibroblasts in the injured liver. Upon activation, they lose retinoid stores and gain contractile and profibrogenic properties, marked by the induction of alpha-smooth muscle actin (α-SMA) [5,11]. Transforming growth factor-β1 (TGF-β1) is a central profibrogenic cytokine that drives HSCs activation and extracellular matrix production across chronic liver diseases, including CHB and CHC [12,13]. Lipid-associated macrophage regulators like CD5L (CD5 antigen-like), also known as AIM (Apoptosis Inhibitor of Macrophage), are emerging as modulators of inflammation and fibrosis through effects on macrophage survival and cytokine signaling, although their role in viral hepatitis is less well defined [14,15]. Glial fibrillary acidic protein (GFAP), traditionally a neural marker, identifies early HSC activation states and stromal remodeling preceding overt fibrosis and is included in the cancer-associated fibroblast biomarkers [16,17].

Despite extensive characterization of liver injury in individual HBV or HCV infection, integrated comparisons of histopathology, immunohistochemical expression of profibrogenic and immune markers, and detailed biochemical profiles in monoinfection vs. CHB+C coinfection are limited. Existing studies have reported differences in necroinflammatory severity and fibrosis between these etiologies, but often in a fragmented manner and without systematic correlation to cellular activation pathways or functional biochemical status [18,19].

The present study aimed to characterize and compare histopathological features, immunohistochemical expression of α-SMA, TGF-β1, CD5L, and GFAP, and biochemical profiles in patients with chronic hepatitis B, chronic hepatitis C, and HBV/HCV coinfection. Furthermore, we investigated the relationship between these molecular and morphological markers and clinically relevant indicators of disease severity, including fibrosis stage and functional liver parameters, in order to identify distinct pathophysiological patterns associated with different viral etiologies.

## 2. Materials and Methods

### 2.1. Study Design and Ethical Considerations

The hospital-based observational study protocol was approved by the Scientific Ethics and Deontology Commission of the University of Medicine and Pharmacy of Craiova (No. 48/29 January 2024) and conducted with strict adherence to the ethical principles of the Declaration of Helsinki.

### 2.2. Study Population

This study included 54 adult patients diagnosed with chronic viral hepatitis, divided as follows: CHB (*n* = 29, 19 females and 10 males), CHC (*n* = 15, 4 females and 11 males), and CHB+C (*n* = 10, 2 females and 8 males). Subjects were recruited from the Gastroenterology service of the Pitești County Emergency Hospital, Argeș County, Romania, for which a liver biopsy was performed.

Inclusion criteria included age over 18 years, confirmed chronic CHB (HBsAg positive ≥ 6 months), CHC (anti-HCV positive, HBV negative), or coinfection CHB+C.

Exclusion criteria included mixed etiologies (alcoholic or drug-induced hepatitis) and active HCC. The biological samples were represented by biopsy liver tissue samples from the Pathological Anatomy Service of the Pitești County Emergency Hospital, Argeș County, Romania.

Given the retrospective nature of the study, data on prior antiviral treatment and disease duration (including newly diagnosed cases) were not uniformly available and could not be reliably assessed.

Demographic, biochemical, and hematological data were collected from electronic records (HIPOCRATE system), including aspartate aminotransferase (AST), alanine aminotransferase (ALT), gamma-glutamyl transferase (GGT), albumin (ALB), total serum bilirubin (TBIL), platelet count (PLT), international normalized ratio (INR), creatinine, total serum cholesterol (CHOL), serum triglycerides (TG), Ishak histological activity and fibrosis scoring system (Ishak) and METAVIR histological grading and staging system (METAVIR).

Using appropriate body mass index (BMI) data, prognostic indices Model for End-Stage Liver Disease (MELD), Model for End-Stage Liver Disease version 3.0 (MELD 3.0) and immunohistochemistry (IHC) H-scores (α-SMA, TGF-β1, CD5L, GFAP) were determined. The data was selected by patient registration number and processed exclusively for research purposes in compliance with the General Data Protection Regulation.

### 2.3. Liver Histology and Histopathological Evaluation

Liver tissue fragments with dimensions of 2–3.5 cm collected by biopsy were fixed in 10% neutral buffered formalin and paraffin-embedded. Serial sections with a thickness of 4 μm were performed and stained with hematoxylin-eosin and Masson’s trichrome for histological observations and evaluation. Masson’s trichrome staining allows the detection of pathological collagen deposits, being the standard for the diagnosis and staging of hepatic fibrosis [20].

Liver fibrosis was assessed using two established ordinal scoring systems, internationally recommended for grading hepatic necroinflammation and the staging of liver fibrosis [21]: the Ishak fibrosis score (stages 0–6) [22] and the METAVIR fibrosis score (stages 0–4) [23].

Histological examination of liver biopsy specimens included the evaluation of portal and lobular inflammatory infiltrates, interface hepatitis, hepatocyte necrosis (isolated and confluent), hepatocyte apoptosis, dystrophic lesions, ballooning degeneration, steatosis, intrahepatocytic pigment, ground glass hepatocytes, Kupffer cell hypertrophy and hyperplasia, sinusoidal changes, biliary duct lesions, portal and septal fibrosis, fibrous bridges, occasional isolation nodules, and cirrhosis. Histological features were recorded using ordinal scales (0–3) or as presence/absence variables, according to standard criteria.

Necroinflammatory activity was quantified using the Ishak Histological Activity Index, which evaluates four distinct components: piecemeal necrosis (A, 0–4), confluent necrosis (B, 0–6), focal necrosis, apoptosis and focal inflammation (C, 0–4), and portal inflammation (D, 0–4). The total necroinflammatory activity score was calculated as the sum of components A–D, with a possible range of 0–18 [22] and reviewed by Theise [24]. In parallel, METAVIR activity scores (0–3) were determined to allow cross-system comparison.

### 2.4. Immunohistochemical Analysis

Serial sections of formalin-fixed, paraffin-embedded liver samples were cut to a thickness of 4 μm. The sections were deparaffinized and then subjected to immunohistochemical staining for the antibodies used, according to the standard laboratory protocol (Table 1).

The selection of these markers in our study is justified by their capacity to characterize key pathogenic mechanisms in chronic viral hepatitis. Specifically, α-SMA highlights HSC activation and phenotypic heterogeneity [25,26,27], while GFAP identifies early and transitional stages of HSCs activation [16,28]. TGF-β1 reflects profibrotic and immunomodulatory signaling pathways that drive fibrosis progression [21,29]. CD5L marks macrophage involvement in chronic inflammation and liver tissue remodeling [14,30,31]. Together, these markers capture central processes underlying the pathogenesis of CHB, CHC and CHB+C.

The IHC expression of the markers used (α-SMA, TGF-β1, CD5L, GFAP) was evaluated semi-quantitatively using the H-score calculated based on the staining intensity and the percentage of positive cells, according to the formula [32] H−score=∑(i×Pi), where *i* = staining intensity evaluated using a predefined semi-quantitative scale (0–3, where 0—absence of staining; 1—weak staining; 2—moderate staining, 3—intense staining), and *Pi* = percentage of positive cells for each intensity.

The H-score ranged from 0 to 300 by combining the staining intensity and the percentage of stained cells at each staining level, thus providing a detailed measure of the overall IHC staining in the tissue sample.

The score was calculated for all 54 samples. For each case, 5 arbitrarily selected fields of view (×200 magnification) were analyzed, avoiding areas of necrosis and artifacts.

For each case, scoring was performed independently by two observers, and the final score represented the mean of the two assessments. Histology and IHC images were acquired using a Nikon Eclipse microscope (Nikon Corporation, Tokyo, Japan) equipped with a digital imaging system, under standardized conditions of illumination, exposure and magnification to ensure inter-sample comparability.

### 2.5. Assessment of Liver Disease Severity

Liver dysfunction severity was primarily evaluated using the MELD 3.0, with the classical MELD score used as a secondary comparative measure. Both scores were treated as continuous variables. Patients were additionally stratified according to Child–Pugh class (A, B, C), considered an ordinal variable reflecting progressive hepatic dysfunction.

### 2.6. Statistical Analysis

Statistical analyses were performed using IBM SPSS Statistics version 27.0.1. Descriptive statistics included mean, standard deviation (SD), median, interquartile range (IQR), minimum, maximum, skewness, and kurtosis. Normality was evaluated with Shapiro–Wilk tests and skew/kurtosis criteria; non-normal variables were described with median and IQR. Group comparisons (CHB, CHC, CHB+C—containing ordinal or non-normal data) used non-parametric Kruskal–Wallis tests, followed by pairwise Mann–Whitney U tests. To account for multiple comparisons, Bonferroni correction was applied for post hoc pairwise analyses following Kruskal–Wallis testing. Given the exploratory nature of the study and the relatively large number of comparisons, results were interpreted cautiously, with additional consideration of effect size estimates. Effect size estimation was performed for non-parametric group comparisons. Eta-squared (η^2^) was calculated using the formula $\eta^{2}=\frac{H\;-\;k\;+\;1}{n\;-\;k}$, where H represents the Kruskal–Wallis statistic, *k* the number of groups, and *n* the total sample size. Effect sizes were interpreted according to conventional thresholds (small ≈ 0.01, moderate ≈ 0.06, large ≥ 0.14).

For variables with non-normal distributions, results are reported as median and interquartile range (IQR), while mean ± SD is additionally provided for comparability with the existing literature.

Associations between ordinal histological variables, functional scores, and age were assessed using Spearman’s rank correlation coefficient (ρ), selected to avoid assumptions of normality and linearity while allowing the evaluation of monotonic relationships. Correlations were calculated for Ishak components, total necroinflammatory activity, METAVIR activity, and patient age.

Spearman’s rank correlation coefficient (ρ) was used to assess the association between IHC marker expression, quantified as H-scores, and liver fibrosis stage. The variables included TGF-β1, α-SMA, and GFAP, correlated against fibrosis stage evaluated on a 0–4 scale (METAVIR-equivalent). The strength of correlations was interpreted according to standard criteria, with values above 0.70 considered strong.

To examine the relationship between marker expression and fibrosis severity, METAVIR stages were recoded into a binary variable: F0–F1 (absent/mild fibrosis, group 0) and F2–F4 (advanced fibrosis, group 1). Non-parametric comparisons were performed using the Mann–Whitney U test.

Principal Component Analysis (PCA) was performed to identify patterns of covariation among biochemical variables. Data suitability for PCA was evaluated using the Kaiser–Meyer–Olkin (KMO) measure of sampling adequacy and Bartlett’s test of sphericity. Variables were standardized prior to analysis. Components were extracted using the principal components method and rotated using Varimax rotation with Kaiser normalization to enhance interpretability. Components with eigenvalues > 1 and loadings > 0.5 were interpreted.

All statistical tests were two-tailed, and statistical significance was defined as *p* < 0.05. The total sample size for all analyses was *N* = 54.

## 3. Results

### 3.1. Clinical and Anthropometric Assessment

Age distributions were broadly comparable across groups. Median ages were 44 years (CHB), 48 years (CHC), and 36 years (CHB+C), closely matching means in CHB and CHB+C, indicating approximately symmetric distributions, while CHC showed a slightly lower mean.

**Figure 1:**
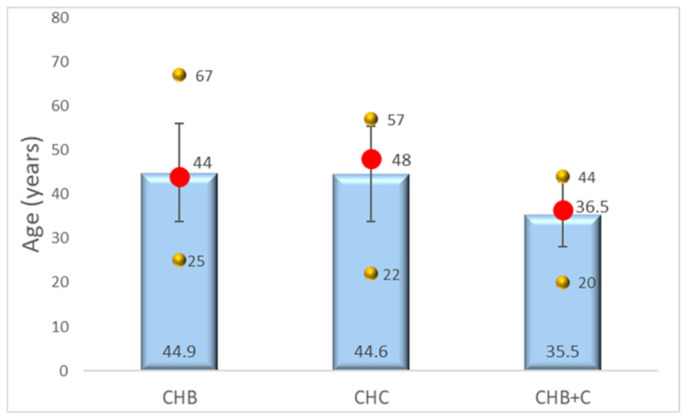
Distribution of age across chronic viral hepatitis etiologies. CHB—chronic hepatitis B, CHC—chronic hepatitis C, CHB+C—chronic hepatitis B+C. Blue columns—the mean age (in years) of patients within each group. Red dots—the median age for each group. Vertical lines—standard deviation (SD) from the mean, indicating the variability of the age data within each group. Upper orange dots—the maximum age within each group. Lower orange dots—the minimum age within the group.

Variance and range were greater in CHB and CHC compared with CHB+C, reflecting higher interindividual variability. Shapiro–Wilk tests confirmed non-significant departures from normality in all groups (*p* > 0.05), with minor asymmetry in CHB+C (Figure 1, Table S1).

**Figure 2:**
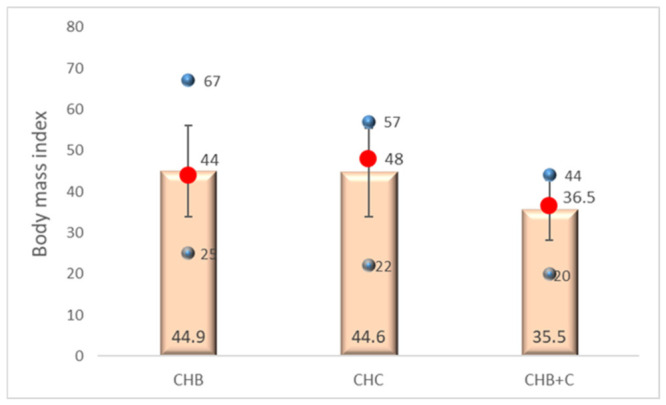
Distribution of body mass index across chronic viral hepatitis etiologies. CHB—chronic hepatitis B, CHC—chronic hepatitis C, CHB+C—chronic hepatitis B+C. Orange columns—the mean BMI within each group. Red dots—the median BMI for each group. Vertical lines—standard deviation (SD) from the mean. Upper blue dots—the maximum BMI within each group. Lower blue dots—the minimum BMI within the group.

BMI distributions showed greater heterogeneity across groups. Median BMI values were 25.32 kg/m^2^ in CHB, 23.64 kg/m^2^ in CHC, and 29.2 kg/m^2^ in CHB+C. Median and interquartile range values revealed substantial dispersion and asymmetry in the CHB+C group (skewness = −1.217) compared with the other groups (Figure 2, Table S1).

Kolmogorov–Smirnov and Shapiro–Wilk tests confirmed non-normality in CHB+C (*p* = 0.040 and *p* = 0.054, respectively), indicating higher BMI heterogeneity among coinfected patients.

### 3.2. Biochemical Profiles

Biochemical alterations varied not only in magnitude but also in their internal consistency across etiological groups. AST and ALT showed marked heterogeneity. In CHB, both enzymes were strongly right-skewed (AST skewness = 4.46; ALT skewness = 2.50) with pronounced kurtosis, indicating the presence of extreme outliers, which is also reflected by medians lower than means. In CHC, AST and ALT remained right-skewed but less markedly so, whereas in CHB+C their distributions were more symmetric, albeit with greater overall dispersion.

GGT followed a similar pattern. In CHB, it showed a clearly non-normal distribution (skewness > 2, kurtosis > 5), while in CHC, it was characterized by wide variability and a flatter distribution. In contrast, CHB+C exhibited more compact and symmetric GGT values, suggesting a more uniform cholestatic profile.

Lipid parameters also differed between groups. CHOL values were approximately normally distributed in CHB and CHB+C, whereas CHC showed right-skewness (1.45) and increased kurtosis (3.81). TG levels were close to normal in CHB and CHC, while CHB+C displayed a flatter distribution (kurtosis = −1.96) with moderate asymmetry.

TBIL in CHB showed a right-skewed, leptokurtic distribution, while CHC and CHB+C were more symmetric. Serum creatinine exhibited marked right-skewness in CHB (skewness = 3.10, kurtosis = 12.17), moderate skewness in CHC, and an approximately symmetric distribution in CHB+C.

Markers of liver synthetic function showed distinct patterns. ALB was non-normally distributed in CHB and CHC, with particularly high kurtosis in CHC (>4), but approached normality in CHB+C. PLT values were approximately normally distributed across all groups. INR showed mild positive skewness in CHB and CHC but remained within acceptable limits (Table 2 and Table S2).

### 3.3. Integrated Biochemical Patterns by Principal Component Analysis (PCA)

**Figure 3:**
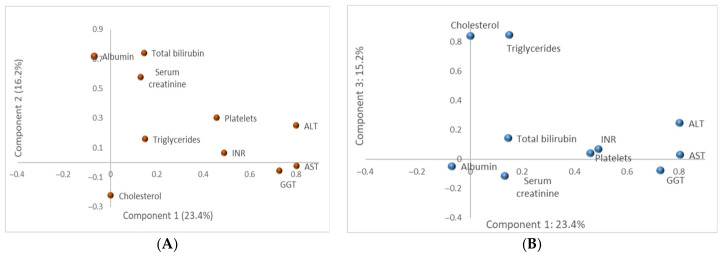
Principal component loading plots of biochemical variables. (**A**) PC1 vs. PC2 loading plot (Component 1, 23.4% and Component 2, 16.2% of explained variance). PC1–PC2 plane highlighting hepatocellular injury and cholestasis-related markers. (**B**) PC1 vs. PC3 loading plot (Component 1, 23.4% and Component 3, 15.2% of explained variance). PC1–PC3 plane highlighting the separation of lipid metabolism markers.

To integrate these variables at a systemic level, Principal Component Analysis (PCA) was applied. The dataset met adequacy criteria (KMO = 0.611; Bartlett’s test χ^2^ = 98.551, df = 45, *p* < 0.001). Three components with eigenvalues > 1 were retained, explaining 54.9% of total variance: Component 1 (hepatocellular injury, 23.4%), Component 2 (hepatic functional impairment, synthetic and excretory axis, 16.2%), and Component 3 (lipid profile, 15.2%). PC1 strongly loaded on AST, ALT, GGT, PLT, and INR; PC2 on ALB, TBIL, and creatinine; and PC3 on CHOL and TG. These components reflect distinct biochemical patterns underlying liver injury, cholestasis, and metabolic alteration (Figure 3A,B, Table S3).

### 3.4. Fibrosis and Necroinflammatory Activity

**Figure 4:**
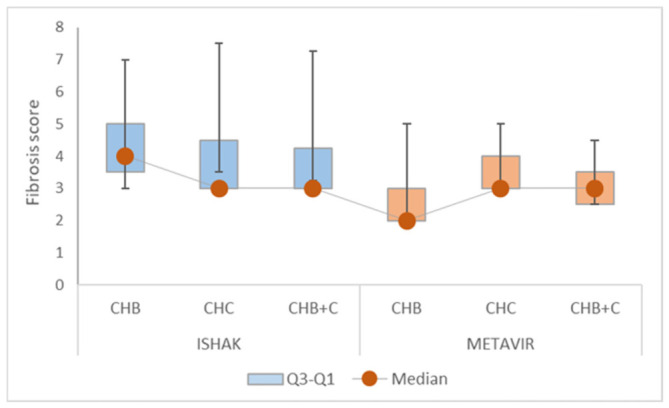
Distribution of Ishak and METAVIR fibrosis scores across chronic viral hepatitis etiologies. CHB—chronic hepatitis B, CHC—chronic hepatitis C, CHB+C—coinfection B+C. Boxplots illustrate the distribution of liver fibrosis severity assessed by the Ishak and Metavir scoring systems. For each group, the box represents the interquartile range (IQR, Q1–Q3), with the lower edge corresponding to the first quartile (Q1) and the upper edge to the third quartile (Q3). The marker within each box indicates the median fibrosis score. Whiskers extend from the box to the minimum and maximum observed values, representing the full range of scores.

Fibrosis severity was moderate-to-advanced across all diagnostic groups. Ishak fibrosis scores clustered between stages 3–5, with broad dispersion and flat distributions (kurtosis < −1), indicating heterogeneity in fibrosis burden. METAVIR staging showed less dispersion, particularly in CHC, consistent with a narrower fibrosis spectrum in this subgroup (Figure 4, Table S4).

Both Ishak and METAVIR fibrosis scores demonstrated significant departures from normality in all groups (Shapiro–Wilk *p* < 0.05), confirming their ordinal and bounded nature.

Spearman correlation analysis demonstrated strong and statistically significant positive associations between all investigated immunohistochemical markers and fibrosis stage (Table 3 and Table S4) (*p* < 0.001 for all comparisons).

Both α-SMA and TGF-β1 showed robust correlations with fibrosis severity (ρ = 0.774 and ρ = 0.756, respectively). GFAP expression also exhibited a strong positive correlation with fibrosis stage (ρ = 0.777). In addition, very strong inter-marker correlations were observed. TGF-β1 expression was highly correlated with α-SMA (ρ = 0.975), while GFAP showed strong associations with both α-SMA and TGF-β1 (ρ = 0.835).

Mann–Whitney U testing revealed that expression levels of TGF-β1, α-SMA, GFAP, and CD5L were significantly higher in patients with advanced fibrosis (F2–F4) compared to those with absent or mild fibrosis (F0–F1). Mean rank values were consistently greater in the advanced fibrosis group (TGF-β1: 28.5 vs. 1.5; α-SMA: 28.5 vs. 1.5; GFAP: 28.5 vs. 1.5; CD5L: 28.5 vs. 1.5), indicating a progressive upregulation of fibrogenic and stellate cell activation markers with disease severity (Table S5).

Necroinflammatory variables showed marked ceiling effects and asymmetric distributions, particularly for METAVIR activity and confluent necrosis, which was absent in all patients. Several parameters exhibited extreme kurtosis, including piecemeal necrosis in CHB+C and portal inflammation in CHC and CHB+C, reflecting clustering of scores rather than true outliers (Table 4 and Table S6).

Normality testing confirmed a consistent departure from normal distribution across most necroinflammatory variables, in line with their ordinal and semi-quantitative nature.

Sex-based comparisons showed no significant differences in any histological parameters, indicating similar necroinflammatory severity between males and females (Table 5 and Table S7), while age-based correlations revealed moderate associations between age and focal necrosis/apoptosis (ρ = 0.420, *p* = 0.002), with limited influence of age on overall necroinflammatory activity (Table 6 and Table S8). Confluent necrosis exhibited zero variance across all groups, precluding meaningful statistical comparison. Spearman correlation revealed that piecemeal necrosis and portal inflammation exhibited strong correlations with total necroinflammatory activity (ρ = 0.812 and 0.886, respectively; *p* < 0.001), indicating that they are primary drivers of overall hepatic injury. Focal necrosis/apoptosis also correlated moderately with total necroinflammatory activity (ρ = 0.721, *p* < 0.001) and METAVIR activity (ρ = 0.389, *p* = 0.004).

Portal necroinflammation subcomponents displayed marked ordinal distributions. MELD 3.0 scores were more normally distributed than classic MELD, particularly in CHB+C, suggesting improved calibration for prognostic assessment (Table 7 and Table S9).

### 3.5. Immunohistochemical Expression Patterns

Median H-scores indicated increasing profibrogenic and injury markers in coinfected patients: TGF-β (CHB 136 → CHB+C 185), CD5L (CHB 135 → CHB+C 197.5), α-SMA (CHB 156 → CHB+C 220), GFAP (CHB 130 → CHB+C 160). Skewness and kurtosis revealed non-normal distributions in CHB and CHC for TGF-β, CD5L, and α-SMA, while GFAP was near-normal in CHB+C (Table 8 and Table S9).

Group comparisons (Kruskal–Wallis analysis) revealed statistically significant differences among diagnostic groups for CD5L H-score (*p* = 0.009), ALT (*p* = 0.002), AST (*p* = 0.011), and GGT (*p* < 0.001). Although statistically significant after Bonferroni correction (*p* = 0.004), this result should be interpreted with caution in the context of multiple comparisons and limited sample size. TGF-β, α-SMA, GFAP H-scores, and Ishak fibrosis did not differ significantly across groups (*p* > 0.05). Although not statistically significant, CHB+C consistently showed higher median ranks for α-SMA, TGF-β, CD5L, and liver enzymes, suggesting a consistent trend toward higher profibrogenic marker expression in CHB+C, although these differences did not reach statistical significance for TGF-β, α-SMA, and GFAP (Table 9 and Table 10, Tables S10 and S11).

Effect size analysis of immunohistochemical markers revealed the heterogeneous magnitudes of group differences. CD5L expression showed a large effect size (η^2^ = 0.146), consistent with its statistically significant variation across etiological groups. In contrast, TGF-β demonstrated a small-to-moderate effect (η^2^ = 0.055), while α-SMA exhibited a small effect size (η^2^ = 0.035).

### 3.6. MELD 3.0 vs. Child–Pugh Validation

MELD 3.0 increased progressively from Child–Pugh A to C, reflecting progressing liver function and better discriminative power than classic MELD, which showed overlapping medians across classes. Spearman correlations confirmed moderate concordance between MELD 3.0 and MELD (ρ = 0.426, *p* = 0.001) and weak negative associations with METAVIR fibrosis (ρ = −0.298, *p* = 0.029). Ishak and METAVIR fibrosis stages correlated strongly (ρ = 0.728, *p* < 0.001), suggesting histological consistency (Table 11 and Table 12 and Table S12).

### 3.7. Histopathological Features in Liver Biopsies

Histological evaluation of the 54 liver biopsy specimens revealed a consistent pattern of portal-dominant inflammation associated with mild lobular involvement, supporting a chronic viral hepatitis phenotype.

**Figure 5:**
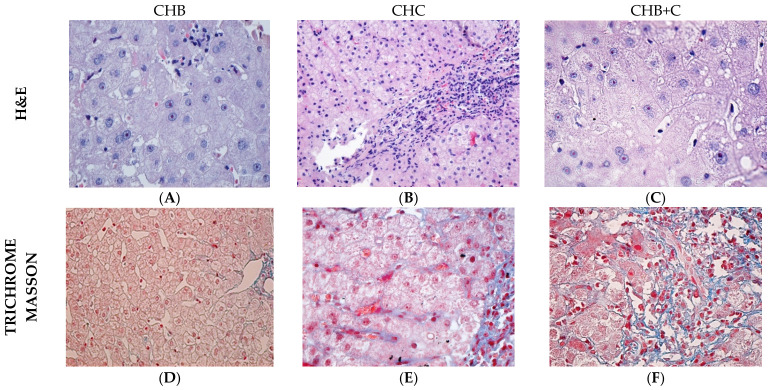
Representative histological images in CHB, CHC and CHB+C. H&E staining (**A**–**C**) to highlight architectural changes and the presence of inflammatory infiltrate and Masson’s Trichrome (**D**–**F**) to assess the degree of hepatic fibrosis. Magnification: ×100 (**B**,**D**); ×400 (**A**,**C**,**E**,**F**).

Portal inflammatory infiltrates were present in nearly all cases and were predominantly moderate, while lobular inflammation remained minimal in most biopsies, indicating relative preservation of lobular architecture (Figure 5B). Interface hepatitis was frequently observed with moderate to marked activity in a substantial proportion of cases, highlighting ongoing periportal injury (Figure 5B).

Markers of hepatocellular injury were common but predominantly non-severe. Isolated intralobular necrosis appeared as rare foci in 66.7% of cases and as multiple foci in 33.3%, while confluent necrosis was absent across all groups, precluding variability-based comparisons. Hepatocyte apoptosis and dystrophic changes were universally present (100%), indicating ongoing chronic cellular stress. Ballooning degeneration was detected in all samples, mainly at mild intensity (Figure 5A).

Steatosis was variable, with minimal (38.9%) and occasional (35.2%) involvement, reflecting heterogeneous lipid accumulation (Figure 5A,C–E). Ground glass hepatocytes were present in 57.4% of cases, consistent with variable HBsAg retention. Biliary duct lesions occurred in 53.7%, while neocanalicular proliferation was less frequent (25.9%).

Fibrotic changes were prominent yet heterogeneous, ranging from fibrous bridging to established cirrhosis in one-third of patients, suggesting that most cases were in advanced pre-cirrhotic or early cirrhotic stages (Figure 5F).

Kupffer cell activation was generally limited, with minimal or focal hypertrophy/hyperplasia in 98.1% of samples. Rare findings such as glycogen nuclei, rosette formation, or mitotic hepatocytes were sporadic.

**Figure 6:**
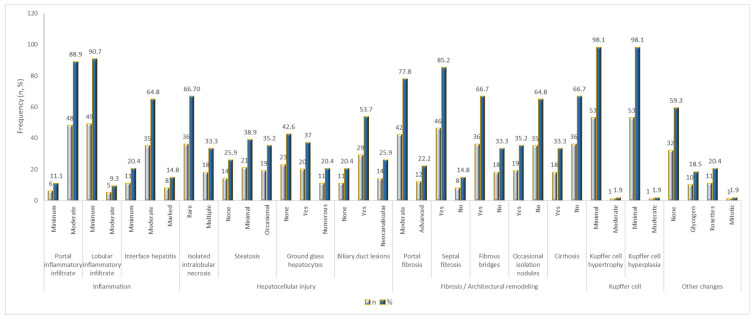
Comparative distribution of histological lesion frequency in chronic viral hepatitis (*N* = 54).

The histological profile is characterized by dominant portal inflammation, persistent low-grade hepatocellular injury, and progressive but heterogeneous fibrotic remodeling, while severe necroinflammatory damage remains uncommon. Synthetic data is displayed in Figure 6 (Table S11).

### 3.8. Comparative Histopathological Features Across CHB, CHC, and CHB+C Groups

Direct comparison between CHB, CHC, and CHB+C revealed distinct patterns of distribution for selected histological variables.

Lobular inflammatory infiltrates were significantly higher in CHC compared with CHB+C (*p* = 0.003), indicating more prominent parenchymal inflammation in HCV-related disease. In contrast, interface hepatitis was most pronounced in CHB+C compared with CHC (*p* = 0.009), suggesting an additive effect of coinfection on portal–periportal injury.

Isolated intralobular necrosis was significantly more severe in CHB than CHC (*p* = 0.027), whereas no differences were observed for confluent necrosis or apoptosis across groups. Ballooning degeneration showed a non-significant trend toward higher values in CHB+C (*p* = 0.127).

Steatosis was significantly more frequent in CHC compared with CHB+C (*p* = 0.005), while ground glass hepatocytes were significantly more prevalent in CHB compared with CHC (*p* < 0.001), consistent with HBV-specific cytoplasmic changes.

Fibrotic parameters showed a trend toward greater severity in CHC, although it was not statistically significant. Portal fibrosis (*p* = 0.059), septal fibrosis (*p* = 0.066), and fibrous bridges (*p* = 0.043) were more prominent in CHC compared with CHB, suggesting a more accelerated fibrogenic pattern in HCV infection.

Other features, including Kupffer cell changes, dystrophic lesions, sinusoidal alterations, and biliary duct lesions, did not differ significantly between groups.

Overall, CHC is characterized by a stronger fibrotic and lobular inflammatory profile, CHB by more prominent HBV-specific cytopathic changes, and CHB+C by enhanced interface hepatitis, reflecting a combined pattern of injury rather than a uniform additive effect (Table 13 and Table S13).

### 3.9. Immunohistochemical Observations

**Figure 7:**
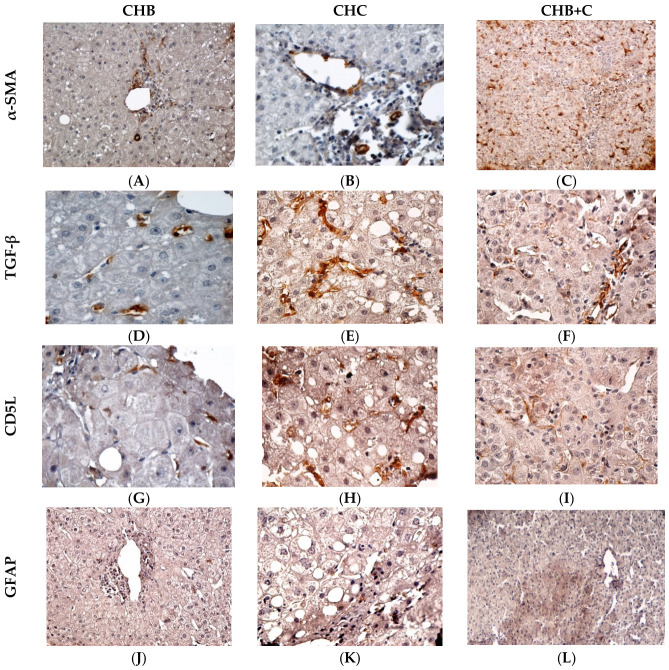
Immunohistochemical expression of α-SMA, TGF-β, CD5L and GFAP in CHB, CHC and CHB+C. Immunostaining for α-SMA (**A**–**C**) highlights the activation of hepatic stellate cells, TGF-β (**D**–**F**) indicates the involvement of profibrotic mechanisms, CD5L (**G**–**I**) reflects the immune response and inflammatory processes, and GFAP (**J**–**L**) marks HSCs in the early stage of activation. Magnification: ×100 (**A**–**C**,**J**,**L**), ×200 (**E**,**F**,**H**,**I**,**K**), ×400 (**D**,**G**).

Immunohistochemical analysis highlighted distinct patterns of fibrogenic activation and immune involvement across CHB, CHC, and CHB+C (Figure 7).

In CHB, α-SMA expression was moderate, being predominantly located at the level of portal spaces in incipient fibrous septa; the fibrotic extension was more limited compared to CHC, in which it was more intense and more extensive, showing a periportal and perisinusoidal distribution, as well as the presence of myofibroblasts in well-defined fibrous ridges. The staining pattern is diffuse, suggesting a sustained activation of HSCs, characteristic of accelerated fibrotic progression, and it may be associated with persistent lobular inflammation. The most intense expression in both the intensity and extension of the α-SMA marker was observed in CHB+C. A broad, periportal and perisinusoidal cell distribution is observed, coexisting with thick, sometimes confluent fibrous septa. The strong staining indicates a marked fibrogenic cell activation and deposition. These features are consistent with a more extensive HSCs activation pattern in coinfection; however, given the lack of statistical significance across groups, this observation should be interpreted cautiously as a descriptive trend.

TGF-β expression in CHB is low to moderate, with predominant secreting cells disseminated in vessel walls in portal and periportal localization, in inflammatory cells, and occasionally in some hepatocytes adjacent to areas of necrosis. The role of TGF-β is rather reactive, and fibrogenesis progresses slowly compared to CHC. The most intense expression of TGF-β, both in terms of intensity and extension, was observed in CHB+C. A wide portal, periportal and perisinusoidal distribution is noted, with intense expression in hepatocytes and stromal cells.

In CHB, CD5L was expressed predominantly in sinusoidal Kupffer cells and less frequently in macrophages of the portal infiltrate. Hepatocytes are generally negative or weakly focally positive. CD5L in CHC was highly expressed in Kupffer cells and macrophages of the portal and periportal infiltrate. Occasionally, weak cytoplasmic signals are observed in hepatocytes, especially in areas of steatosis. We also noted its distribution to be diffuse, extensive, and accentuated in cells inside the fibrous septa, in the periportal areas, and in the cells in areas with persistent chronic inflammation. In CHB+C, CD5L expression was marked in Kupffer cells and in portal and septal macrophages. Focal expression is possible in periportal hepatocytes and occasionally in activated sinusoidal endothelial cells. The distribution was diffuse and confluent, of increased intensity, suggesting a spatially expanded macrophage activation in coinfection, supported by the statistically significant CD5L expression and an accelerated fibrotic process (Tables S13 and S14).

In CHB, GFAP was expressed in perisinusoidal stellate cells, predominantly in portal and lobular areas with active inflammation. The distribution is focal or discontinuous, reflecting early HSC activation in the context of chronic inflammation, without extensive fibrotic remodeling. In CHC, GFAP expression was marked in perisinusoidal and periportal HSCs. Occasionally, it can be expressed in mesenchymal cells in thin fibrous septa. The distribution is diffuse, with lobular extension, indicating widespread activation of HSCs and their involvement in stromal remodeling and fibrogenesis.

In comparison, in CHB+C, intense GFAP expression was observed in perisinusoidal HSCs and periportal and septal stellate cells (possible myofibroblast cells) with extension into fibrous septa and areas of bridging fibrosis. Its distribution, corroborated by a marked loss of normal lobular architecture, reflects extensive HSC activation and an accelerated fibrotic process compared with monoinfections.

## 4. Discussions

### 4.1. Clinical and Anthropometric Characteristics

Our analysis suggests modest demographic differences between groups, with coinfected patients (CHB+C) tending to be younger, possibly reflecting distinct exposure patterns and epidemiological context [10,33,34,35,36,37,38,39]. However, these differences are unlikely to be major drivers of the observed fibrogenic profiles [10,33,34,35,36,37,38,39].

In contrast, the higher BMI observed in the CHB+C group may be more relevant from a pathophysiological perspective. Metabolic factors are known to interact with inflammatory and fibrogenic pathways, potentially enhancing HSC activation and accelerating fibrosis progression [40,41,42,43]. This interaction may be particularly important in coinfected patients, where viral and host-related factors converge to shape disease severity.

BMI differences further support the role of host metabolic status as a disease modifier. CHB+C patients showed higher median BMI and a non-normal distribution, suggesting the clustering of overweight or obese individuals. Elevated BMI is a known risk factor for HCC across multiple liver diseases [40] and may act as a cofactor enhancing fibrogenesis and necroinflammatory activity. These findings support integrated management strategies targeting both viral and metabolic components [41]. The heterogeneity of BMI in CHC reinforces the need for careful adjustment of metabolic confounders in multivariable analyses, as metabolic status may modulate disease expression [42].

### 4.2. Biochemical Profiles and Principal Component Analysis

Non-invasive fibrosis markers, including AST/ALT ratio, ALBI score, GPR, APRI, FIB-4, INPR and FibroQ, have been extensively investigated as surrogate tools for assessing liver disease severity [10]. In our previous study, we performed a comprehensive evaluation of these biomarkers and demonstrated their discriminative performance across different etiologies of chronic liver disease.

In the present study, biochemical markers showed marked non-normal distributions, particularly in CHB patients, reflecting episodic hepatocellular injury and heterogeneity of disease activity. CHB+C patients displayed more symmetric distributions but higher mean values for ALT, GGT, TG, PLT, and TBIL, suggesting a more homogeneous but globally intensified biochemical phenotype.

PCA identified three distinct biochemical patterns, capturing 54.9% of total variance. Sampling adequacy was moderate (KMO = 0.611), indicating an acceptable but not optimal structure for dimension reduction. Bartlett’s test confirmed statistical suitability for factor extraction (*p* < 0.001). Component 1 (AST, ALT, GGT, PLT, INR) reflects hepatocellular injury and inflammatory necroactivity. Component 2 (ALB, TBIL, creatinine) reflects impaired synthetic and excretory function. Component 3 (CHOL, TG) reflects metabolic dysregulation.

From a clinical perspective, these components provide an integrated view of disease expression beyond isolated biomarkers. Component 1 aligns with necroinflammatory activity and shows concordance with fibrosis-associated histological changes, while Component 2 reflects progressive functional impairment consistent with advanced disease stages. Component 3 separates metabolic involvement, suggesting a partially independent metabolic signature in chronic viral hepatitis.

Taken together, PCA not only reduces dimensionality but also highlights distinct but overlapping functional domains of liver injury, which may support future efforts in patient stratification based on dominant biochemical phenotypes rather than single-analyte interpretation.

Elevated liver enzymes and biochemical markers observed in CHB+C patients are consistent with previous reports indicating more pronounced hepatocellular injury in coinfection compared with monoinfection. Similar increases in AST, ALT, TBIL, and creatinine have been described in cohorts from different populations, supporting the association between coinfection and more severe biochemical disturbance. These alterations likely reflect enhanced hepatocellular damage and accelerated fibrogenesis, contributing to a higher risk of progression to cirrhosis and hepatocellular carcinoma [43,44,45,46].

Comparable biochemical patterns have also been reported in other chronic liver disease contexts, including HCC and viral coinfections, where elevated transaminases and bilirubin levels correlate with increased tissue injury and disease severity [33,47]. Overall, the close association between enzymatic profiles and clinical severity supports their role as accessible biomarkers for disease monitoring, particularly in settings where advanced diagnostic tools are limited [10,48].

### 4.3. Histological Fibrosis and Necroinflammatory Activity

Histological scores, including Ishak and METAVIR fibrosis stages and necroinflammatory activity, are used for grading and staging chronic liver diseases [49]. Their non-normal distribution reflects ordinal structure and ceiling effects inherent to staging systems.

MELD 3.0 showed improved stability compared to classic MELD, particularly in CHB+C, supporting its utility in heterogeneous cohorts.

Sex-based analysis revealed no significant differences in necroinflammatory features, suggesting that histological severity is independent of sex and supporting uniform clinical stratification.

Age showed only weak associations with focal necrosis/apoptosis, indicating that hepatocyte injury is largely age-independent, consistent with pediatric data [50]. However, fibrosis has been associated with age in specific HBV subgroups [51], indicating context-dependent effects.

The correlation between fibrosis and immunological indices (CD3+, CD4+, CD8+, CD16+, CD19+) suggests that immune activity contributes to structural progression [52].

### 4.4. Immunohistochemical H-Scores and Coinfection Effects

Non-parametric Kruskal–Wallis testing ensured robust handling of non-normal distributions. Post hoc comparisons demonstrated significantly higher CD5L, AST, ALT, and GGT in CHB+C vs. monoinfected patients, corroborating both immunohistochemical and biochemical evidence of increased liver injury and fibrosis. These markers align with higher fibrosis stages and underscore their potential as semi-quantitative indicators of disease progression and for patient stratification in clinical studies. H-score analysis revealed elevated CD5L expression and liver enzymes in CHB+C patients [30], suggesting synergistic profibrogenic and inflammatory responses in coinfection. However, given the current level of evidence, CD5L should be interpreted as a component of the inflammatory microenvironment rather than a direct driver of fibrosis, and further studies are needed to clarify its precise mechanistic contribution. Although TGF-β, α-SMA, and GFAP did not reach statistical significance, their higher ranks in co-infected patients indicate a trend toward enhanced fibrogenic activation. HBV/HCV coinfection is associated with a stronger profibrogenic response than either monoinfection, characterized by increased activation of the TGF-β1 pathway. Coinfected hepatocytes show higher TGF-β1 expression and enhanced induction of downstream fibrogenic genes compared to HBV or HCV alone, indicating a trend toward amplified fibrogenic signaling. This effect is further reinforced in hepatocyte– HSCs coculture systems, where coinfection promotes greater HSCs activation, migration, and invasion. The data suggest that the higher TGF-β-driven signaling observed in HBV/HCV coinfection reflects a synergistic mechanism leading to accelerated fibrogenesis, mediated through OCT4/Nanog-dependent pathways [53].

α-SMA expression, a marker of HSCs activation, shows a strong positive correlation with fibrosis severity in CHB. Increased α-SMA immunoreactivity across periportal, perisinusoidal, and pericentral regions parallels higher fibrosis scores, indicating intensified fibrogenic activation [54,55]. In the context of CHB/CHC coinfection, higher α-SMA ranks would therefore be consistent with enhanced HSCs activation and a more pronounced fibrogenic response, rather than with viral dominance, per se. In CHC, α-SMA expression likewise increases with advancing fibrosis, reflecting progressive HSCs activation. In contrast, GFAP expression is highest in early disease stages and declines as fibrosis progresses, supporting its role as a marker of early HSCs activation rather than advanced fibrogenesis [28]. Applied to CHB/CHC coinfection, elevated α-SMA combined with altered GFAP expression would be more indicative of a shift from early to fully activated HSC’sphenotypes, rather than a parallel upregulation of both markers.

### 4.5. Functional Scores and Correlations with Histology

Although Child–Pugh and MELD scores are primarily validated in cirrhotic patients, they are also used in chronic liver disease to estimate hepatic functional reserve and to stratify disease severity in research settings. MELD 3.0 exhibited a clear monotonic increase across Child–Pugh classes, with increasing dispersion in Child–Pugh C, reflecting advanced cirrhosis heterogeneity. Classic MELD showed overlapping medians, supporting prior concerns regarding its reduced sensitivity [56]. The weak negative association observed between MELD 3.0 and METAVIR fibrosis highlights the intrinsic difference between structural and functional assessment of chronic liver disease. While METAVIR reflects histological fibrosis staging, MELD 3.0 is primarily a composite marker of hepatic functional reserve and systemic physiological dysfunction. This dissociation may be particularly evident in compensated or early decompensated disease, where substantial architectural fibrosis may coexist with relatively preserved synthetic function and stable laboratory parameters [57,58]. Consequently, MELD 3.0 does not directly mirror fibrosis burden but rather captures the clinical impact of hepatic dysfunction, explaining the observed weak inverse relationship. In contrast, the strong correlation between Ishak and METAVIR confirms the robustness of histological fibrosis assessment across scoring systems [59,60].

Spearman correlation demonstrates a strong association between key immunohistochemical markers and fibrosis severity, supporting their relevance in liver fibrogenesis. The strong positive correlation between α-SMA expression and fibrosis stage confirms its role as a reliable indicator of HSCs activation and profibrogenic signaling [61,62]. GFAP expression also showed a strong positive correlation with fibrosis stage. suggesting that HSCs activation markers may persist during disease progression, reflecting ongoing cellular activation [28,63,64].

The extremely high correlation between TGF-β1 and α-SMA further supports a mechanistic link between cytokine signaling and HSCs activation, indicating that fibrogenesis is driven by a coordinated molecular network rather than isolated pathways [59,60]. The strong association of GFAP with both markers reinforces the concept of a dynamic continuum of HSCs activation states [59,61].

From a pathophysiological perspective, these results provide direct evidence that histopathological and immunohistochemical alterations are closely aligned with structural liver damage, as reflected by fibrosis staging [62]. This directly addresses the relationship between tissue-level changes and disease severity, emphasizing that the activation of fibrogenic pathways is not merely present but quantitatively linked to fibrosis progression [59].

A critical implication of these findings is that multiple markers, rather than a single indicator, may be required to fully characterize the complexity of HSCs activation and fibrogenesis [28]. The concurrent elevation of α-SMA, TGF-β1, and GFAP suggests overlapping but not identical roles, highlighting the need for integrated interpretation in both research and clinical contexts [60].

CD5L showed a parallel increase with fibrosis, indicating a potential link between macrophage activation and fibrogenesis and supporting the contribution of inflammatory signaling to disease progression [63]. Interestingly, CD5L, although primarily associated with inflammatory processes rather than direct fibrogenesis, showed a parallel increase, potentially highlighting crosstalk between inflammation and fibrotic progression. These results provide a clear histopathological correlation with clinical fibrosis staging, reinforcing the utility of these markers in assessing disease severity. As limitations, in the present study, the F0–F1 group contained only two patients, limiting statistical robustness for this comparison. Future studies should include larger early-stage cohorts to confirm these findings.

From a mechanistic perspective, the observed associations between TGF-β, α-SMA, GFAP, and CD5L can be interpreted within established fibrogenic signaling networks. TGF-β is a central upstream regulator of HSCs activation, primarily acting through SMAD-dependent signaling pathways that promote the transcriptional activation of extracellular matrix genes and α-SMA expression [64,65]. This cascade represents a key driver of transdifferentiation of quiescent HSCs into contractile, myofibroblast-like cells. In parallel, macrophage-associated signaling, reflected by CD5L expression, may contribute to the maintenance of a pro-inflammatory microenvironment through cytokine-mediated amplification of TGF-β signaling and sustained HSCs activation. GFAP expression reflects an early phenotypic stage of HSCs activation, preceding full myofibroblastic transformation, and may indicate ongoing cellular plasticity within the fibrogenic niche. Together, these pathways suggest a coordinated interaction between inflammatory and fibrogenic signaling networks rather than isolated molecular events.

In our study, the combined histopathological and immunohistochemical findings support a model in which liver fibrogenesis is driven by interconnected processes involving HSCs activation, profibrogenic cytokine signaling, and macrophage-mediated inflammation. Importantly, the absence of significant correlations between these markers and MELD 3.0 indicates dissociation between structural remodeling and functional impairment.

### 4.6. Histopathological Features

Biopsies showed a characteristic spectrum of chronic viral hepatitis, dominated by portal and periportal inflammation and ongoing hepatocyte injury. Apoptosis and dystrophic lesions were universal, while ballooning degeneration and mild steatosis reflected hepatocyte stress without extensive fat accumulation. Hepatocyte ballooning reflects cytoskeletal damage and imbalanced protein homeostasis, but the causal mechanism remains unclear [66].

Ground-glass hepatocytes were present in a subset of cases, consistent with chronic HBV antigen accumulation. Fibrotic remodeling was evident, with portal fibrosis in ~78% and septal fibrosis in 85% of cases, while cirrhosis was limited to one-third of patients. Kupffer cell changes were minimal. These detailed observations underscore the value of median and IQR reporting for ordinal histological metrics, enabling robust correlation with clinical and biochemical parameters.

The histological patterns observed align with the distinct pathophysiology of HBV and HCV infections [67]. Portal inflammation appears as a common baseline feature, unaffected by viral etiology, whereas lobular inflammation is exacerbated in CHC, reflecting the virus’s predilection for hepatocyte parenchyma [66,68]. The pronounced ground glass hepatocytes in CHB biopsies underscore HBV’s characteristic cytoplasmic viral inclusions.

Coinfection (CHB+C) showed an intermediate histological profile, with increased interface hepatitis, suggesting additive effects of the two viruses on hepatocellular injury. Steatosis was more prominent in CHC, likely reflecting both viral and host-related metabolic influences, consistent with genotype-dependent lipid accumulation [69]. Fibrosis, particularly portal and septal, was more advanced in CHC, in line with sustained lobular inflammation and supported by the higher frequency of fibrous bridges. Other histological features did not differ significantly between groups, suggesting lower sensitivity to viral etiology or later-stage manifestation.

In summary, distinct etiological patterns were observed, as follows: CHB was associated with cytoplasmic changes and focal necrosis, CHC with lobular inflammation and fibrogenesis, while CHB+C combined features of both, particularly at the interface level, supporting the relevance of integrated histopathological evaluation for understanding disease progression and clinical stratification. These findings should be interpreted primarily as comparative differences between disease groups, as no healthy reference tissue was available.

Integration analysis further revealed that CHB+C patients consistently showed higher profibrogenic and hepatocellular injury markers across biochemical and immunohistochemical assessments. PCA delineated distinct biochemical patterns corresponding to histological severity. Fibrosis and necroinflammatory scores correlated with Component 1 markers, while MELD 3.0 provided improved prognostic stratification compared with classic MELD, particularly in advanced disease stages [70,71].

### 4.7. Immunohistochemical Findings

Immunohistochemical analysis demonstrated consistent activation of HSCs across all groups, with α-SMA expression increasing from CHB to CHC and reaching the highest levels in CHB+C. The predominantly portal and perisinusoidal distribution supports its role as a marker of fibrogenic activation, closely associated with necroinflammatory activity and fibrosis progression [72,73,74]. These findings are consistent with previous studies showing that α-SMA expression correlates with fibrosis severity and reflects the expansion of activated HSC populations during chronic liver injury [75,76].

TGF-β expression showed a similar pattern, with more intense and diffuse staining in CHC and maximal expression in CHB+C. Its localization in hepatocytes, inflammatory, and stromal cells supports its central role in driving fibrogenesis through HSC activation and extracellular matrix accumulation. The enhanced expression observed in coinfection further suggests amplification of profibrogenic signaling pathways and accelerated fibrotic remodeling [64,65,66,67,68,69,70,71,72,73,74,75,76,77].

CD5L expression was predominantly localized in hepatic macrophages, with stronger and more diffuse staining in CHC and CHB+C. This pattern indicates active macrophage involvement in inflammatory and fibrogenic processes, supporting its proposed role as a mediator linking immune activation and fibrosis progression. The distribution, mainly in sinusoidal Kupffer cells, suggests that CD5L reflects localized macrophage activation rather than diffuse fibrotic remodeling [10,14,54,78].

GFAP is classically expressed by astrocytes in the nervous system, but in the liver, it is an early marker of HSC in quiescent or early activation stages. It highlights the cytoplasmic network of HSC and is useful in assessing early stromal remodeling [79].

In our study, immunohistochemical analysis of GFAP revealed a progressive activation of HSCs, with focal expression in CHB, diffuse distribution in CHC and extensive overexpression in CHB+C, supporting their central role in hepatic fibrogenesis. IHC studies on liver biopsies in CHC show that GFAP marks an early stage of HSC activation, before the expression of other fibrotic markers such as α-SMA [28], being expressed in perisinusoidal areas.

In CHC, Carotti et al. [16] show that α-SMA-positive HSCs in smooth muscle grow and expand throughout the parenchyma as fibrosis progresses. In parallel, GFAP-positive HSCs are more evenly distributed in early stages and confined to the periphery of the lobule in advanced stages of fibrosis. Thus, GFAP expressions begin to decrease as fibrosis progresses, at which point α-SMA becomes more highly expressed.

From an integrated perspective, the strong correlations observed between inflammatory and fibrogenic markers (α-SMA, TGF-β, GFAP) and fibrosis stages support the existence of a coordinated fibrogenic network driven by HSC activation. In this context, CD5L may represent a complementary component of the inflammatory microenvironment, potentially linking macrophage-mediated immune responses to fibrogenic signaling pathways.

From a clinical standpoint, these findings suggest that the combined assessment of inflammatory and fibrogenic markers may contribute to a more refined characterization of disease activity in chronic viral hepatitis. However, their direct clinical applicability remains limited at present, and these results should be interpreted as exploratory.

Prospective validation in larger, longitudinal cohorts is required to determine their potential role in risk stratification and clinical decision-making.

However, this study has several limitations that should be considered when interpreting the results.

First, the study is based on a single-center cohort, which may limit the generalizability of the findings due to potential center-specific patient selection and management practices. In addition, external validation in an independent cohort is not available; therefore, the results should be regarded as hypothesis-generating rather than definitive.

Second, the relatively small size of the CHB+C and CHC groups likely reduced the statistical power and increased the risk of type II error. This is particularly relevant for immunohistochemical comparisons, where several markers showed consistent directional trends without reaching statistical significance. Effect size analysis indicated a small-to-moderate effect for TGF-β and a small effect for α-SMA, suggesting that limited power may partly explain the lack of statistical significance, although small effect sizes also indicate that true differences may be modest.

Third, the absence of detailed treatment histories represents an additional limitation. Prior or ongoing antiviral therapy may influence inflammatory activity, fibrosis progression, and immunohistochemical marker expression, potentially acting as a confounding factor that could not be controlled in the present analysis.

Fourth, the relatively large number of statistical comparisons increases the risk of type I error. Although Bonferroni correction was applied for post hoc analyses, this approach does not fully eliminate the possibility of false positive findings, particularly in the context of multiple endpoints and small subgroup sizes.

Finally, patient selection may introduce bias, as the inclusion of cases with available liver biopsy may preferentially capture patients with more advanced disease.

Despite these limitations, the monocentric design ensured a high degree of methodological consistency, particularly in histopathological scoring and immunohistochemical evaluation, reducing inter-observer and inter-laboratory variability. This strengthens the internal validity of the observed patterns. Overall, the study provides an integrated multi-parametric dataset that supports the need for larger, prospective, multicenter studies with standardized clinical and therapeutic data.

## 5. Conclusions

This study demonstrates that CHB+C coinfection does not merely intensify histological scores but induces a qualitative reorganization of the hepatic fibrogenic and inflammatory response. Our results indicate a consistent pattern of increased profibrogenic and inflammatory marker expression in coinfected patients. However, as most immunohistochemical differences did not reach statistical significance, this pattern should be interpreted as suggestive rather than conclusive evidence of a synergistic effect.

α-SMA expression highlights a progressive gradient from CHB to CHC, reaching its maximum intensity and spatial extension in CHB+C. In coinfected livers, the diffuse periportal and perisinusoidal distribution of activated myofibroblasts, together with thick and confluent fibrous septa, indicates sustained and widespread HSCs activation rather than a localized or reactive fibrotic response. This pattern reflects an accelerated fibrogenic trajectory with a high risk of architectural distortion and progression toward cirrhosis.

TGF-β immunoreactivity further supports this interpretation. While CHB shows a limited, mainly reactive expression pattern and CHC displays a more pronounced profibrotic involvement, CHB+C is characterized by intense and extensive TGF-β expression in both parenchymal and stromal compartments. This suggests that coinfection promotes a permissive microenvironment for continuous collagen deposition and impaired extracellular matrix turnover, integrating inflammation and fibrosis into a self-sustaining loop.

The immune component, assessed by CD5L expression, reveals that macrophage activation is not only increased in coinfection but also spatially reorganized. In CHB+C, the diffuse and confluent distribution of CD5L-positive Kupffer cells and portal macrophages, including their presence within fibrous septa, indicates a synergistic immune activation that actively participates in fibrogenesis rather than serving a purely inflammatory role.

GFAP expression completes this profile by demonstrating that HSC in coinfected patients is activated early, extensively and persistently. The involvement of perisinusoidal, periportal and septal stellate cells, together with their extension into bridging fibrosis, confirms that coinfection affects not only the intensity but also the timing and coordination of stromal remodeling.

Taken together, these findings suggest that CHB+C coinfection may represent a distinct and potentially more complex pathogenic profile, characterized by an integrated fibrogenic–inflammatory response rather than the simple superposition of two monoinfections. However, this interpretation should be considered exploratory, given the limited sample size and the lack of consistent statistical significance across all parameters. The coinfected liver coordinated alterations involving HSCs activation, profibrotic cytokine signaling and macrophage-mediated immune responses, which may help explain the more aggressive clinical course observed in these patients, but these observations require validation in larger, independent cohorts.

The histopathological and immunohistochemical profile of liver involvement is complemented by serological, biochemical, and hematological data, providing a comprehensive overview of health status in CHB, CHC, and CHB+C.

This integrated perspective has important implications for disease stratification and therapeutic decision-making, emphasizing the need to consider coinfection as a specific biological entity rather than an additive variant of chronic viral hepatitis.

## Figures and Tables

**Table 1:** Immunohistochemical antibodies and protocols applied for the evaluation of fibrogenic, inflammatory and stellate cell markers.

Antibody	Dilution	Source/Catalogue	Antigen Unmasking Method	Amplification Method
α-SMA Clone 1A4 Monoclonal	1:10	DAKO IR 611 (Glostrup, Denmark)	Microwave 5 cycles × 4 min Citrate buffer pH = 6	HISTOFINE DAB-3S
TGF-β1—TB21 Monoclonal IgG1	1:10	Santa Cruz Biotechnology SC-52893 (Heidelberg, Germany)	Microwave 5 cycles × 4 min Citrate buffer pH = 6	HISTOFINE DAB-3S
CD5L (AIM) Polyclonal IgG	1:10	Sigma-Aldrich HPA068384 (Stockholm, Sweden)	Microwave 5 cycles × 4 min Citrate buffer pH = 6	HISTOFINE DAB-3S
GFAP Polyclonal	1:10	DAKO IR524 (Glostrup, Denmark)	Microwave 5 cycles × 4 min Citrate buffer pH = 6	HISTOFINE DAB-3S

**Table 2:** Descriptive statistics and distributional characteristics of biochemical variables by diagnosis.

Variable	Group	Mean ± SD	Median (IQR)	Variance	Range	Skewness	Kurtosis	Distribution Note
AST (U/L)	CHB	34.17 ± 27.85	27.0 (12.5)	775.79	18–170	4.46	21.77	Strongly non-normal, extreme outliers
	CHC	34.00 ± 11.75	32.0 (11.0)	138.14	24–61	1.56	1.80	Right-skewed
	CHB+C	44.00 ± 11.34	45.5 (18.25)	128.67	22–60	−0.51	0.07	Approx. normal
ALT (U/L)	CHB	34.86 ± 19.43	29.0 (23.5)	377.55	15–114	2.50	9.14	Non-normal
	CHC	30.00 ± 17.57	22.0 (22.0)	308.71	16–77	1.74	2.78	Non-normal
	CHB+C	67.70 ± 30.14	74.5 (52.0)	908.68	23–110	−0.18	−1.54	Broad, symmetric
GGT (U/L)	CHB	27.34 ± 20.33	18.0 (18.0)	413.23	11–101	2.23	5.54	Non-normal
	CHC	52.27 ± 38.03	30.0 (65.0)	1445.92	19–119	0.88	−1.18	High dispersion
	CHB+C	55.40 ± 10.28	54.5 (16.25)	105.60	36–70	−0.29	0.02	Approx. normal
CHOL (mg/dL)	CHB	207.45 ± 29.67	210.0 (41.5)	880.33	139–268	−0.11	−0.03	Normal
	CHC	190.60 ± 40.50	181.0 (48.0)	1639.97	128–304	1.45	3.81	Right-skewed
	CHB+C	179.70 ± 34.93	176.5 (60.5)	1219.79	126–235	0.10	−0.82	Normal
TG (mg/dL)	CHB	96.90 ± 21.01	95.0 (29.5)	441.45	63–149	0.39	−0.11	Normal
	CHC	99.73 ± 34.31	98.0 (51.0)	1177.21	40–149	−0.21	−0.71	Normal
	CHB+C	124.60 ± 34.08	137.0 (64.5)	1161.16	76–164	−0.32	−1.96	Flat distribution
ALB (g/dL)	CHB	2.82 ± 0.60	2.60 (0.65)	0.36	2.1–4.4	1.33	1.29	Mild non-normal
	CHC	2.51 ± 0.48	2.50 (0.60)	0.23	1.8–3.9	1.70	4.81	Non-normal
	CHB+C	3.25 ± 0.44	3.35 (0.83)	0.20	2.6–3.8	−0.23	−1.83	Near-normal
PLT (×10^3^/µL)	CHB	215.93 ± 61.18	216.0 (66.5)	3742.99	74–359	−0.04	0.39	Normal
	CHC	209.47 ± 58.81	203.0 (86.0)	3459.12	135–350	0.94	0.87	Approx. normal
	CHB+C	281.20 ± 65.67	282.5 (72.5)	4312.84	165–390	−0.42	0.35	Normal
INR	CHB	1.05 ± 0.12	1.05 (0.13)	0.014	0.88–1.32	0.90	0.80	Normal
	CHC	1.00 ± 0.07	1.00 (0.08)	0.004	0.89–1.16	0.56	1.31	Normal
	CHB+C	1.12 ± 0.19	1.20 (0.25)	0.035	0.80–1.40	−0.36	−0.69	Normal
TBIL (mg/dL)	CHB	0.85 ± 0.31	0.85 (0.30)	0.093	0.44–1.93	1.65	4.45	Non-normal
	CHC	0.80 ± 0.24	0.80 (0.40)	0.059	0.40–1.20	0.11	−0.62	Normal
	CHB+C	1.29 ± 0.33	1.30 (0.63)	0.108	0.80–1.80	−0.03	−1.06	Normal
Creatinine (mg/dL)	CHB	0.86 ± 0.22	0.82 (0.17)	0.048	0.63–1.80	3.10	12.17	Strongly non-normal
	CHC	0.91 ± 0.25	0.80 (0.40)	0.062	0.60–1.50	1.06	0.70	Mild non-normal
	CHB+C	1.01 ± 0.16	1.00 (0.32)	0.025	0.80–1.20	0.00	−1.59	Normal

Notes: Median (IQR) is emphasized for non-normal distributions, while mean ± SD is provided for comparability. Skewness and kurtosis indicate heterogeneous distributions.

**Table 3:** Spearman correlation matrix between immunohistochemical markers (TGF-β1, α-SMA, GFAP) and liver fibrosis stage (METAVIR 0–4).

Correlations	TGF-β1	α-SMA	GFAP	Fibrosis Stage 0–4
TGF-β1	1.000	0.975 **	0.835 **	0.756 **
α-SMA	0.975 **	1.000	0.835 **	0.774 **
GFAP	0.835 **	0.835 **	1.000	0.777 **
Fibrosis stage 0–4	0.756 **	0.774 **	0.777 **	1.000

** Correlation is significant at the 0.01 level (2-tailed). Color scale: correlations range from green (ρ = 1, strongest) to light yellow (lower ρ values).

**Table 4:** Necroinflammatory activity scores by diagnosis.

Variable	Group	Mean ± SD	Median (IQR)	Range	Skewness	Kurtosis	Distribution
Piecemeal necrosis	CHB	2.83 ± 0.71	3.0 (1.0)	1–4	−0.38	0.47	Mild non-normal
	CHC	2.67 ± 0.49	3.0 (1.0)	2–3	−0.79	−1.62	Non-normal
	CHB+C	3.00 ± 0.47	3.0 (0.0)	2–4	0.00	4.50	Highly peaked
Focal necrosis/apoptosis	CHB	2.34 ± 0.55	2.0 (1.0)	1–3	−0.01	−0.72	Approx. normal
	CHC	2.00 ± 0.00	2.0 (0.0)	2–2	-	-	Constant
	CHB+C	2.20 ± 0.42	2.0 (0.25)	2–3	1.78	1.41	Right-skewed
Portal inflammation	CHB	2.48 ± 0.57	3.0 (1.0)	1–3	−0.54	−0.66	Non-normal
	CHC	2.40 ± 0.51	2.0 (1.0)	2–3	0.46	−2.09	Flat
	CHB+C	2.60 ± 0.52	3.0 (1.0)	2–3	−0.48	−2.28	Flat
Total necroinflammatory activity	CHB	7.66 ± 1.59	9.0 (3.0)	3–9	−0.94	0.64	Left-skewed
	CHC	7.07 ± 0.80	7.0 (2.0)	6–8	−0.13	−1.35	Flat
	CHB+C	7.80 ± 1.14	8.0 (1.25)	6–10	0.48	0.55	Approx. normal
METAVIR activity	CHB	1.48 ± 0.57	2.0 (1.0)	0–2	−0.54	−0.66	Non-normal
	CHC	2.00 ± 0.00	2.0 (0.0)	2–2	-	-	Constant
	CHB+C	2.00 ± 0.00	2.0 (0.0)	2–2	-	-	Constant
Histologic Activity Index (HAI)	CHB	7.66 ± 1.70	9.0 (2.5)	3–10	−0.88	0.27	Mild asymmetry
	CHC	7.20 ± 1.32	7.0 (1.0)	5–10	0.22	0.65	Approx. normal
	CHB+C	7.60 ± 0.84	8.0 (1.0)	6–9	−0.39	0.37	Approx. normal

Notes: Median (IQR) is emphasized for non-normal distributions, while mean ± SD is provided for comparability. Skewness and kurtosis indicate heterogeneous distributions.

**Table 5:** Ishak and METAVIR activity scores by sex (*n* = 40 with known sex).

Histological Feature	Mean ± SD	Min–Max	Median	Mann–Whitney U	Z	*p*-Value
Piecemeal necrosis	2.81 ± 0.62	1–4	3	356.0	−0.134	0.894
Confluent necrosis	0.00 ± 0.00	0–0	0	362.5	0.000	1.000
Focal necrosis/apoptosis	2.22 ± 0.46	1–3	2	294.5	−1.549	0.121
Portal inflammation	2.48 ± 0.54	1–3	2	356.5	−0.119	0.905
Total necroinflammatory activity	7.52 ± 1.34	3–10	8	325.0	−0.672	0.502
METAVIR activity	1.72 ± 0.49	0–2	2	318.5	−1.002	0.316

Notes: Values are expressed as mean ± standard deviation (SD), median, and minimum–maximum range. Comparisons between male and female patients were performed using the Mann–Whitney U test for ordinal and non-normally distributed variables. Z values correspond to standardized test statistics. All tests were two-tailed. Statistical significance was set at *p* < 0.05. Variables with zero variance were reported descriptively and excluded from inferential interpretation.

**Table 6:** Spearman correlations among necroinflammatory components and age.

Feature	Piecemeal Necrosis	Confluent Necrosis	Focal Necrosis/ Apoptosis	Portal Inflammation	Total Necroinflammatory Activity	METAVIR Activity	Age
Piecemeal necrosis	1	0	0.311 *	0.578 **	0.812 **	0.449 **	−0.007
Confluent necrosis	0	1	0	0	0	0	0
Focal necrosis/apoptosis	0.311 *	0	1	0.595 **	0.721 **	0.389 **	0.42 **
Portal inflammation	0.578 **	0	0.595 **	1	0.886 **	0.615 **	0.156
Total necroinflammatory activity	0.812 **	0	0.721 **	0.886 **	1	0.582 **	0.196
METAVIR activity	0.449 **	0	0.389 **	0.615 **	0.582 **	1	0.119
Age	−0.007	0	0.42 **	0.156	0.196	0.119	1

**: Correlation is significant at the 0.01 level (2-tailed). *: Correlation is significant at the 0.05 level (2-tailed). Color scale: correlations range from green (ρ = 1, strongest) to yellow (lower ρ values).

**Table 7:** Prognostic scores MELD vs. MELD 3.0 by diagnosis.

Score	Group	Mean ± SD	Median (IQR)	Range	Skewness	Kurtosis	Distribution
MELD 3.0	CHB	19.03 ± 1.57	19.0 (2.0)	16–24	0.95	2.39	Right-skewed
	CHC	18.67 ± 1.63	19.0 (1.0)	16–23	1.08	2.81	Right-skewed
	CHB+C	18.80 ± 1.99	18.0 (4.0)	17–22	0.56	−1.60	Approx. normal
MELD	CHB	7.48 ± 1.68	7.0 (1.5)	6–14	2.35	7.31	Highly skewed
	CHC	7.07 ± 1.10	7.0 (2.0)	6–10	1.34	2.45	Non-normal
	CHB+C	9.30 ± 1.57	9.0 (3.0)	7–12	0.46	−0.59	Approx. normal

Notes: Median (IQR) is emphasized for non-normal distributions, while mean ± SD is provided for comparability. Skewness and kurtosis indicate heterogeneous distributions.

**Table 8:** Descriptive statistics of H-scores by etiology.

Variable	Diagnosis	Median (IQR)	Mean ± SD	Range	Skewness	Kurtosis
TGF-β H-score	CHB	136.0 (101.0)	162.4 ± 53.7	65–232	0.082	−1.415
	CHC	175.0 (55.0)	189.3 ± 40.5	120–240	−0.385	−1.051
	CHB+C	185.0 (62.5)	190.5 ± 37.0	135–240	0.007	−0.794
CD5L H-score	CHB	135.0 (87.5)	148.3 ± 46.4	50–205	−0.196	−0.974
	CHC	175.0 (60.0)	181.7 ± 44.5	100–230	−0.545	−0.963
	CHB+C	197.5 (40.0)	198.0 ± 26.4	155–235	−0.309	−0.430
α-SMA H-score	CHB	156.0 (108.0)	180.0 ± 57.1	55–246	−0.209	−0.918
	CHC	215.0 (40.0)	207.7 ± 43.3	115–250	−1.364	1.189
	CHB+C	220.0 (45.0)	217.5 ± 33.9	155–255	−0.939	0.213
GFAP H-score	CHB	130.0 (86.5)	146.4 ± 47.0	45–210	−0.188	−0.703
	CHC	155.0 (20.0)	151.3 ± 17.2	120–175	−0.934	−0.162
	CHB+C	160.0 (15.0)	162.0 ± 9.8	145–180	0.187	0.480

Notes: Median (IQR) is emphasized for non-normal distributions, while mean ± SD is provided for comparability. Skewness and kurtosis indicate heterogeneous distributions.

**Table 9:** Group comparisons for H-scores and biochemical variables.

Variable	Diagnosis	*N*	K-W H	*p*-Value
Ishak Fibrosis	CHB	29	1.446	0.485
	CHC	15		
	CHB+C	10		
TGF-β H-score	CHB	29	4.798	0.091
	CHC	15		
	CHB+C	10		
CD5L H-score	CHB	29	9.458	0.009
	CHC	15		
	CHB+C	10		
α-SMA H-score	CHB	29	3.764	0.152
	CHC	15		
	CHB+C	10		
GFAP H-score	CHB	29	2.071	0.355
	CHC	15		
	CHB+C	10		
AST (U/L)	CHB	29	8.998	0.011
	CHC	15		
	CHB+C	10		
ALT (U/L)	CHB	29	12.791	0.002
	CHC	15		
	CHB+C	10		
GGT (U/L)	CHB	29	17.157	0.000
	CHC	15		
	CHB+C	10		

**Table 10:** Post hoc pairwise comparisons (Bonferroni-adjusted).

Variable	Group 1	Group 2	*N* (G1/G2)	Mean Rank (G1/G2)	*p*-Value (2-Tailed)
CD5L H-score	CHB	CHC	29/15	19.22/28.83	0.018
	CHB	CHB+C	29/10	17.28/27.90	0.011
	CHC	CHB+C	15/10	12.20/14.20	0.503
AST (U/L)	CHB	CHC	29/15	20.83/25.73	0.229
	CHB	CHB+C	29/10	16.98/28.75	0.004
	CHC	CHB+C	15/10	10.47/16.80	0.036
ALT (U/L)	CHB	CHC	29/15	24.34/18.93	0.184
	CHB	CHB+C	29/10	16.84/29.15	0.002
	CHC	CHB+C	15/10	9.07/18.90	0.001
GGT (U/L)	CHB	CHC	29/15	18.67/29.90	0.006
	CHB	CHB+C	29/10	16.02/31.55	0.000
	CHC	CHB+C	15/10	11.33/15.50	0.165

**Table 11:** Distribution of MELD 3.0 and MELD across Child–Pugh classes.

Score	Child–Pugh	*N*	Median (IQR)	Mean ± SD	Range	Skewness	Kurtosis
MELD 3.0	A	3	17.0 (–)	16.67 ± 0.58	16–17	−1.73	-
	B	29	18.0 (1.0)	18.14 ± 1.09	16–21	0.77	0.86
	C	22	20.0 (2.0)	20.18 ± 1.40	19–24	1.36	1.54
MELD	A	3	7.0 (–)	7.33 ± 0.58	7–8	1.73	-
	B	29	7.0 (2.0)	7.66 ± 1.49	6–11	0.92	−0.06
	C	22	7.0 (2.25)	7.82 ± 2.04	6–14	1.75	3.35

Note: Data are presented as median and interquartile range (IQR) as robust descriptors for non-normal distributions. Mean ± standard deviation (SD) is additionally reported to facilitate comparability with previously published studies. Skewness and kurtosis values are provided to quantify deviations from a Gaussian distribution and to justify the use of non-parametric statistical approaches. The range is included to illustrate the extent of clinical variability, which is particularly relevant in advanced stages of liver disease.

**Table 12:** Spearman correlation among MELD scores and histological fibrosis scores in patients with chronic viral hepatitis.

Spearman’s Rho	MELD 3.0	MELD	ISHAK Fibrosis	METAVIR Fibrosis
MELD 3.0	1.000	0.426 **	−0.156	−0.298 *
MELD	0.426 **	1.000	−0.109	−0.166
ISHAK Fibrosis	−0.156	−0.109	1.000	0.728 **
METAVIR Fibrosis	−0.298 *	−0.166	0.728 **	1.000

**: Correlation is significant at the 0.01 level (2-tailed). *: Correlation is significant at the 0.05 level (2-tailed). Color scale: correlations range from yellow (ρ = 1, strongest) to green (lower ρ values).

**Table 13:** Histopathological features in liver biopsies across CHB, CHC, and CHB+C patients (*N* = 54).

Feature	Median (IQR)	Kruskal–Wallis H	*p*-Value	Significant Differences (*p* < 0.05)	Interpretation
Portal inflammatory infiltrate	1 (1–1)	5.711	0.058	NS	Similar across groups
Lobular inflammatory infiltrate	2 (2–2)	23.796	<0.001	CHC > CHB+C	More prominent lobular inflammation in CHC
Interface hepatitis	2 (2–2)	8.912	0.012	CHB+C > CHC	Highest in coinfection
Isolated intralobular hepatocyte necrosis	1 (1–2)	11.742	0.003	CHB > CHC	More severe in CHB
Intralobular confluent hepatocyte necrosis	0 (0–0)	0.000	1.000	NS	No differences between groups
Hepatocyte apoptosis	1 (1–2)	1.558	0.459	NS	Evenly distributed
Dystrophic lesions	1 (1–1)	0.000	1.000	NS	No differences
Ballooning degeneration	1 (1–1)	9.593	0.008	CHB+C > CHB	Slightly higher in coinfection
Steatosis	1 (0–2)	6.249	0.044	CHC > CHB+C	More frequent in CHC
Intrahepatocyte pigment	0 (0–0)	0.000	1.000	NS	No differences
Ground glass hepatocytes	0 (0–1)	24.483	<0.001	CHB > CHC	Typical HBV cytoplasmic changes
Kupffer cell hypertrophy	1 (1–1)	4.400	0.111	NS	Minor differences
Kupffer cell hyperplasia	1 (1–1)	4.400	0.111	NS	Minor differences
Portal fibrosis	1 (1–1)	7.108	0.029	CHC > CHB	More fibrosis in CHC
Septal fibrosis	1 (1–1)	7.946	0.019	CHC > CHB	More fibrosis in CHC
Fibrous bridges	1 (0–1)	6.295	0.043	CHC > CHB	More advanced fibrosis in CHC
Other features	-	-	-	NS	No significant differences

Note: Median (IQR) = median and interquartile range; Kruskal–Wallis H = non-parametric test comparing three groups; *p*-value = significance of Kruskal–Wallis test; significant differences = result of pairwise Mann–Whitney U tests where *p* < 0.05. NS = not significant.

## Data Availability

The original contributions presented in this study are included in the article/Supplementary Materials. Further inquiries can be directed to the corresponding author.

## Supplementary Materials

The following supporting information can be downloaded at: https://www.mdpi.com/article/10.3390/diseases14050165/s1, Table S1: Age and BMI by diagnostics (descriptive statistics—explore); Table S2: Biochemical variables by diagnosis (descriptive statistics—explore); Table S3: Principal Component Analysis; Table S4: Spearman correlation; Table S5: Mann–Whitney U test; Table S6: Fibrosis staging by diagnosis (descriptive statistics—explore); Table S7: Ishak necroinflammatory scores system—Sex-Based Analysis; Table S8: Ishak necroinflammatory scores system—Age-Based Correlation Analysis; Table S9: H-score (descriptive statistics—explore); Table S10: Kruskal-Wallis Test; Table S11: Post-hoc Pairwise Comparisons; Table S12: Distribution of MELD 3.0 across Child–Pugh classes; Table S13: Frequencies of histological features; Table S14: Comparative histological features across CHB, CHC, and CHB+C groups.

## Abbreviations

The following abbreviations are used in this manuscript:

AIM	Apoptosis Inhibitor of Macrophage
ALB	Serum Albumin
ALT	Alanine Aminotransferase
AST	Aspartate Aminotransferase
BMI	Body Mass Index
CD5L	CD5 Antigen-Like
CHB	Chronic Hepatitis B
CHB+C	Coinfection with Hepatitis B and C Viruses
CHC	Chronic Hepatitis C
CHOL	Total Serum Cholesterol
GFAP	Glial Fibrillary Acidic Protein
GGT	Gamma-Glutamyl Transferase
HBV	Hepatitis B Virus
HCC	Hepatocellular Carcinoma
HCV	Hepatitis C Virus
HSC	Hepatic Stellate Cells
IHC	Immunohistochemistry
INR	International Normalized Ratio
IQR	Interquartile Range
ISHAK	Ishak Histological Activity and Fibrosis Scoring System
MELD	Model for End-Stage Liver Disease
MELD 3.0	Model for End-Stage Liver Disease, Version 3.0
METAVIR	METAVIR Histological Grading and Staging System
PCA	Principal Component Analysis
PLT	Platelet Count
SD	Standard Deviation
TBIL	Total Serum Bilirubin
TG	Serum Triglycerides
TGF-β1	Transforming Growth Factor Beta 1
α-SMA	Alpha-Smooth Muscle Actin
ρ (rho)	Spearman’s Rank Correlation Coefficient
