# Supplementary material for: A Unified Histopathological Framework of Liver Fibrogenesis in Chronic Viral Hepatitis B, C and Coinfection
Source: Diseases. 2026 May 8;14(5):165. doi: 10.3390/diseases14050165 (PMC13205463; doi:10.3390/diseases14050165)
Supplement: Supplementary file 1 [file diseases-14-00165-s001.zip › diseases-4272763-supplementary.pdf]

Supplementary Material

Integrated Histopathological, Immunohistochemical and Biochemical Assessment of Chronic Hepatitis B, C and HBV/HCV Coinfection

Table S1. Age and BMI by diagnostics (descriptive statistics – explore)

Case Processing Summary

		Cases					
		Valid		Missing		Total	
	CHB, CHC, CHB+C	N	Percent	N	Percent	N	Percent
Age	CHB	29	100.0%	0	0.0%	29	100.0%
	CHC	15	100.0%	0	0.0%	15	100.0%
	CHB+C	10	100.0%	0	0.0%	10	100.0%
BMI	CHB	29	100.0%	0	0.0%	29	100.0%
	CHC	15	100.0%	0	0.0%	15	100.0%
	CHB+C	10	100.0%	0	0.0%	10	100.0%

Descriptives

	CHB, CHC, CHB+C			Statistic	Std. Error
Age	CHB	Mean		44.8966	2.06623
		95% Confidence Interval for Mean	Lower Bound	40.6641	
			Upper Bound	49.1290	
		5% Trimmed Mean		44.7567	
		Median		44.0000	
		Variance		123.810	
		Std. Deviation		11.12701	
		Minimum		25.00	
		Maximum		67.00	
		Range		42.00	
		Interquartile Range		19.50	
		Skewness		.254	.434
		Kurtosis		-.912	.845

	CHC	Mean		44.6000	2.77712
		95% Confidence Interval for Mean	Lower Bound	38.6437	
			Upper Bound	50.5563	
		5% Trimmed Mean		45.1667	
		Median		48.0000	
		Variance		115.686	
		Std. Deviation		10.75573	
		Minimum		22.00	
		Maximum		57.00	
		Range		35.00	
		Interquartile Range		15.00	
		Skewness		-.920	.580
		Kurtosis		-.130	1.121
	CHB+C	Mean		35.5000	2.30097
		95% Confidence Interval for Mean	Lower Bound	30.2949	
			Upper Bound	40.7051	
		5% Trimmed Mean		35.8889	
		Median		36.5000	
		Variance		52.944	
		Std. Deviation		7.27629	
		Minimum		20.00	
		Maximum		44.00	
		Range		24.00	
		Interquartile Range		10.25	
		Skewness		-1.096	.687
		Kurtosis		1.062	1.334
BMI	CHB	Mean		24.8607	.72007
		95% Confidence Interval for Mean	Lower Bound	23.3857	
			Upper Bound	26.3357	
		5% Trimmed Mean		24.8348	
		Median		25.3200	
		Variance		15.036	

		Std. Deviation		3.87768	
		Minimum		17.42	
		Maximum		32.54	
		Range		15.12	
		Interquartile Range		5.96	
		Skewness		.027	.434
		Kurtosis		-.369	.845
	CHC	Mean		23.7607	.96272
		95% Confidence Interval for Mean	Lower Bound	21.6958	
			Upper Bound	25.8255	
		5% Trimmed Mean		23.7835	
		Median		23.6400	
		Variance		13.903	
		Std. Deviation		3.72861	
		Minimum		18.46	
		Maximum		28.65	
		Range		10.19	
		Interquartile Range		6.86	
		Skewness		.030	.580
		Kurtosis		-1.740	1.121
	CHB+C	Mean		28.2000	1.58416
		95% Confidence Interval for Mean	Lower Bound	24.6164	
			Upper Bound	31.7836	
		5% Trimmed Mean		28.4389	
		Median		29.2000	
		Variance		25.096	
		Std. Deviation		5.00955	
		Minimum		18.50	
		Maximum		33.60	
		Range		15.10	
		Interquartile Range		6.07	
		Skewness		-1.217	.687

	Kurtosis	.572	1.334

Tests of Normality

	CHB, CHC, CHB+C	Kolmogorov-Smirnov[a] Statistic	df	Sig.	Shapiro-Wilk Statistic	df	Sig.
Age	CHB	.106	29	.200*	.963	29	.380
	CHC	.218	15	.052	.901	15	.099
	CHB+C	.173	10	.200*	.922	10	.372
BMI	CHB	.076	29	.200*	.982	29	.891
	CHC	.178	15	.200*	.894	15	.076
	CHB+C	.268	10	.040	.848	10	.054

*. This is a lower bound of the true significance. a. Lilliefors Significance Correction

Table S2. Biochemical variables by diagnosis (descriptive statistics – explore)

Case Processing Summary

		Cases					
		Valid		Missing		Total	
	CHB, CHC, CHB+C	N	Percent	N	Percent	N	Percent
AST(U/L)	CHB	29	100.0%	0	0.0%	29	100.0%
	CHC	15	100.0%	0	0.0%	15	100.0%
	CHB+C	10	100.0%	0	0.0%	10	100.0%
ALT (U/L)	CHB	29	100.0%	0	0.0%	29	100.0%
	CHC	15	100.0%	0	0.0%	15	100.0%
	CHB+C	10	100.0%	0	0.0%	10	100.0%
GGT (U/L)	CHB	29	100.0%	0	0.0%	29	100.0%
	CHC	15	100.0%	0	0.0%	15	100.0%
	CHB+C	10	100.0%	0	0.0%	10	100.0%
CHOL (mg/dL)	CHB	29	100.0%	0	0.0%	29	100.0%
	CHC	15	100.0%	0	0.0%	15	100.0%
	CHB+C	10	100.0%	0	0.0%	10	100.0%
TG (mg/dL)	CHB	29	100.0%	0	0.0%	29	100.0%
	CHC	15	100.0%	0	0.0%	15	100.0%
	CHB+C	10	100.0%	0	0.0%	10	100.0%
ALB (g/dL)	CHB	29	100.0%	0	0.0%	29	100.0%
	CHC	15	100.0%	0	0.0%	15	100.0%
	CHB+C	10	100.0%	0	0.0%	10	100.0%
PLT	CHB	29	100.0%	0	0.0%	29	100.0%
	CHC	15	100.0%	0	0.0%	15	100.0%
	CHB+C	10	100.0%	0	0.0%	10	100.0%
INR	CHB	29	100.0%	0	0.0%	29	100.0%

	CHC	15	100.0%	0	0.0%	15	100.0%
	CHB+C	10	100.0%	0	0.0%	10	100.0%
TBIL	CHB	29	100.0%	0	0.0%	29	100.0%
	CHC	15	100.0%	0	0.0%	15	100.0%
	CHB+C	10	100.0%	0	0.0%	10	100.0%
creatinina serica	CHB	29	100.0%	0	0.0%	29	100.0%
	CHC	15	100.0%	0	0.0%	15	100.0%
	CHB+C	10	100.0%	0	0.0%	10	100.0%

Descriptives

	CHB, CHC, CHB+C			Statistic	Std. Error
AST(U/L)	CHB	Mean		34.1724	5.17218
		95% Confidence Interval for Mean	Lower Bound	23.5777	
			Upper Bound	44.7671	
		5% Trimmed Mean		29.3180	
		Median		27.0000	
		Variance		775.791	
		Std. Deviation		27.85302	
		Minimum		18.00	
		Maximum		170.00	
		Range		152.00	
		Interquartile Range		12.50	
		Skewness		4.460	.434
		Kurtosis		21.765	.845
	CHC	Mean		34.0000	3.03472
		95% Confidence Interval for Mean	Lower Bound	27.4912	
			Upper Bound	40.5088	
		5% Trimmed Mean		33.0556	
		Median		32.0000	
		Variance		138.143	
		Std. Deviation		11.75342	
		Minimum		24.00	
		Maximum		61.00	
		Range		37.00	
		Interquartile Range		11.00	

		Skewness		1.560	.580
		Kurtosis		1.804	1.121
	CHB+C	Mean		44.0000	3.58701
		95% Confidence Interval for Mean	Lower Bound	35.8856	
			Upper Bound	52.1144	
		5% Trimmed Mean		44.3333	
		Median		45.5000	
		Variance		128.667	
		Std. Deviation		11.34313	
		Minimum		22.00	
		Maximum		60.00	
		Range		38.00	
		Interquartile Range		18.25	
		Skewness		-.508	.687
		Kurtosis		.070	1.334
ALT (U/L)	CHB	Mean		34.8621	3.60819
		95% Confidence Interval for Mean	Lower Bound	27.4710	
			Upper Bound	42.2531	
		5% Trimmed Mean		32.5862	
		Median		29.0000	
		Variance		377.552	
		Std. Deviation		19.43069	
		Minimum		15.00	
		Maximum		114.00	
		Range		99.00	
		Interquartile Range		23.50	
		Skewness		2.502	.434
		Kurtosis		9.144	.845
	CHC	Mean		30.0000	4.53662
		95% Confidence Interval for Mean	Lower Bound	20.2699	
			Upper Bound	39.7301	
		5% Trimmed Mean		28.1667	
		Median		22.0000	

		Variance		308.714	
		Std. Deviation		17.57027	
		Minimum		16.00	
		Maximum		77.00	
		Range		61.00	
		Interquartile Range		22.00	
		Skewness		1.740	.580
		Kurtosis		2.782	1.121
	CHB+C	Mean		67.7000	9.53246
		95% Confidence Interval for Mean	Lower Bound	46.1361	
			Upper Bound	89.2639	
		5% Trimmed Mean		67.8333	
		Median		74.5000	
		Variance		908.678	
		Std. Deviation		30.14428	
		Minimum		23.00	
		Maximum		110.00	
		Range		87.00	
		Interquartile Range		52.00	
		Skewness		-.182	.687
		Kurtosis		-1.540	1.334
GGT (U/L)	CHB	Mean		27.3448	3.77484
		95% Confidence Interval for Mean	Lower Bound	19.6124	
			Upper Bound	35.0772	
		5% Trimmed Mean		24.7126	
		Median		18.0000	
		Variance		413.234	
		Std. Deviation		20.32816	
		Minimum		11.00	
		Maximum		101.00	
		Range		90.00	
		Interquartile Range		18.00	
		Skewness		2.229	.434

				Statistic	Std. Error
		Kurtosis		5.541	.845
	CHC	Mean		52.2667	9.81809
		95% Confidence Interval for Mean	Lower Bound	31.2090	
			Upper Bound	73.3244	
		5% Trimmed Mean		50.4074	
		Median		30.0000	
		Variance		1445.924	
		Std. Deviation		38.02530	
		Minimum		19.00	
		Maximum		119.00	
		Range		100.00	
		Interquartile Range		65.00	
		Skewness		.877	.580
		Kurtosis		-1.175	1.121
	CHB+C	Mean		55.4000	3.24962
		95% Confidence Interval for Mean	Lower Bound	48.0489	
			Upper Bound	62.7511	
		5% Trimmed Mean		55.6667	
		Median		54.5000	
		Variance		105.600	
		Std. Deviation		10.27619	
		Minimum		36.00	
		Maximum		70.00	
		Range		34.00	
		Interquartile Range		16.25	
		Skewness		-.291	.687
		Kurtosis		.020	1.334
CHOL (mg/dL)	CHB	Mean		207.4483	5.50964
		95% Confidence Interval for Mean	Lower Bound	196.1623	
			Upper Bound	218.7343	
		5% Trimmed Mean		207.5077	
		Median		210.0000	
		Variance		880.328	

		Std. Deviation		29.67031	
		Minimum		139.00	
		Maximum		268.00	
		Range		129.00	
		Interquartile Range		41.50	
		Skewness		-.106	.434
		Kurtosis		-.030	.845
	CHC	Mean		190.6000	10.45617
		95% Confidence Interval for Mean	Lower Bound	168.1738	
			Upper Bound	213.0262	
		5% Trimmed Mean		187.7778	
		Median		181.0000	
		Variance		1639.971	
		Std. Deviation		40.49656	
		Minimum		128.00	
		Maximum		304.00	
		Range		176.00	
		Interquartile Range		48.00	
		Skewness		1.451	.580
		Kurtosis		3.805	1.121
	CHB+C	Mean		179.7000	11.04441
		95% Confidence Interval for Mean	Lower Bound	154.7158	
			Upper Bound	204.6842	
		5% Trimmed Mean		179.6111	
		Median		176.5000	
		Variance		1219.789	
		Std. Deviation		34.92548	
		Minimum		126.00	
		Maximum		235.00	
		Range		109.00	
		Interquartile Range		60.50	
		Skewness		.097	.687
		Kurtosis		-.821	1.334

TG (mg/dL)	CHB	Mean		96.8966	3.90161
		95% Confidence Interval for Mean	Lower Bound	88.9045	
			Upper Bound	104.8886	
		5% Trimmed Mean		96.2126	
		Median		95.0000	
		Variance		441.453	
		Std. Deviation		21.01079	
		Minimum		63.00	
		Maximum		149.00	
		Range		86.00	
		Interquartile Range		29.50	
		Skewness		.392	.434
		Kurtosis		-.112	.845
	CHC	Mean		99.7333	8.85893
		95% Confidence Interval for Mean	Lower Bound	80.7328	
			Upper Bound	118.7338	
		5% Trimmed Mean		100.3148	
		Median		98.0000	
		Variance		1177.210	
		Std. Deviation		34.31049	
		Minimum		40.00	
		Maximum		149.00	
		Range		109.00	
		Interquartile Range		51.00	
		Skewness		-.206	.580
		Kurtosis		-.712	1.121
	CHB+C	Mean		124.6000	10.77569
		95% Confidence Interval for Mean	Lower Bound	100.2237	
			Upper Bound	148.9763	
		5% Trimmed Mean		125.1111	
		Median		137.0000	
		Variance		1161.156	
		Std. Deviation		34.07573	

		Minimum		76.00	
		Maximum		164.00	
		Range		88.00	
		Interquartile Range		64.50	
		Skewness		-.321	.687
		Kurtosis		-1.963	1.334
ALB (g/dL)	CHB	Mean		2.8172	.11142
		95% Confidence Interval for Mean	Lower Bound	2.5890	
			Upper Bound	3.0455	
		5% Trimmed Mean		2.7692	
		Median		2.6000	
		Variance		.360	
		Std. Deviation		.60004	
		Minimum		2.10	
		Maximum		4.40	
		Range		2.30	
		Interquartile Range		.65	
		Skewness		1.329	.434
		Kurtosis		1.291	.845
	CHC	Mean		2.5133	.12300
		95% Confidence Interval for Mean	Lower Bound	2.2495	
			Upper Bound	2.7772	
		5% Trimmed Mean		2.4759	
		Median		2.5000	
		Variance		.227	
		Std. Deviation		.47640	
		Minimum		1.80	
		Maximum		3.90	
		Range		2.10	
		Interquartile Range		.60	
		Skewness		1.702	.580
		Kurtosis		4.806	1.121
	CHB+C	Mean		3.2500	.14004

		95% Confidence Interval for Mean	Lower Bound	2.9332	
			Upper Bound	3.5668	
		5% Trimmed Mean		3.2556	
		Median		3.3500	
		Variance		.196	
		Std. Deviation		.44284	
		Minimum		2.60	
		Maximum		3.80	
		Range		1.20	
		Interquartile Range		.83	
		Skewness		-.225	.687
		Kurtosis		-1.831	1.334
PLT	CHB	Mean		215.9310	11.36084
		95% Confidence Interval for Mean	Lower Bound	192.6594	
			Upper Bound	239.2027	
		5% Trimmed Mean		215.8678	
		Median		216.0000	
		Variance		3742.995	
		Std. Deviation		61.18002	
		Minimum		74.00	
		Maximum		359.00	
		Range		285.00	
		Interquartile Range		66.50	
		Skewness		-.042	.434
		Kurtosis		.386	.845
	CHC	Mean		209.4667	15.18579
		95% Confidence Interval for Mean	Lower Bound	176.8964	
			Upper Bound	242.0369	
		5% Trimmed Mean		205.7963	
		Median		203.0000	
		Variance		3459.124	
		Std. Deviation		58.81432	
		Minimum		135.00	

		Maximum		350.00	
		Range		215.00	
		Interquartile Range		86.00	
		Skewness		.939	.580
		Kurtosis		.868	1.121
	CHB+C	Mean		281.2000	20.76739
		95% Confidence Interval for Mean	Lower Bound	234.2209	
			Upper Bound	328.1791	
		5% Trimmed Mean		281.6111	
		Median		282.5000	
		Variance		4312.844	
		Std. Deviation		65.67225	
		Minimum		165.00	
		Maximum		390.00	
		Range		225.00	
		Interquartile Range		72.50	
		Skewness		-.416	.687
		Kurtosis		.349	1.334
INR	CHB	Mean		1.0534	.02203
		95% Confidence Interval for Mean	Lower Bound	1.0083	
			Upper Bound	1.0986	
		5% Trimmed Mean		1.0483	
		Median		1.0500	
		Variance		.014	
		Std. Deviation		.11863	
		Minimum		.88	
		Maximum		1.32	
		Range		.44	
		Interquartile Range		.13	
		Skewness		.900	.434
		Kurtosis		.795	.845
	CHC	Mean		1.0020	.01713
			Lower Bound	.9653	

		95% Confidence Interval for Mean	Upper Bound	1.0387	
		5% Trimmed Mean		.9994	
		Median		1.0000	
		Variance		.004	
		Std. Deviation		.06635	
		Minimum		.89	
		Maximum		1.16	
		Range		.27	
		Interquartile Range		.08	
		Skewness		.557	.580
		Kurtosis		1.305	1.121
	CHB+C	Mean		1.1200	.05925
		95% Confidence Interval for Mean	Lower Bound	.9860	
			Upper Bound	1.2540	
		5% Trimmed Mean		1.1222	
		Median		1.2000	
		Variance		.035	
		Std. Deviation		.18738	
		Minimum		.80	
		Maximum		1.40	
		Range		.60	
		Interquartile Range		.25	
		Skewness		-.360	.687
		Kurtosis		-.692	1.334
TBIL	CHB	Mean		.8510	.05667
		95% Confidence Interval for Mean	Lower Bound	.7349	
			Upper Bound	.9671	
		5% Trimmed Mean		.8236	
		Median		.8500	
		Variance		.093	
		Std. Deviation		.30520	
		Minimum		.44	

		Maximum		1.93	
		Range		1.49	
		Interquartile Range		.30	
		Skewness		1.648	.434
		Kurtosis		4.446	.845
	CHC	Mean		.8000	.06249
		95% Confidence Interval for Mean	Lower Bound	.6660	
			Upper Bound	.9340	
		5% Trimmed Mean		.8000	
		Median		.8000	
		Variance		.059	
		Std. Deviation		.24202	
		Minimum		.40	
		Maximum		1.20	
		Range		.80	
		Interquartile Range		.40	
		Skewness		.105	.580
		Kurtosis		-.624	1.121
	CHB+C	Mean		1.2900	.10376
		95% Confidence Interval for Mean	Lower Bound	1.0553	
			Upper Bound	1.5247	
		5% Trimmed Mean		1.2889	
		Median		1.3000	
		Variance		.108	
		Std. Deviation		.32813	
		Minimum		.80	
		Maximum		1.80	
		Range		1.00	
		Interquartile Range		.63	
		Skewness		-.031	.687
		Kurtosis		-1.056	1.334
creatinina serica	CHB	Mean		.8603	.04063
			Lower Bound	.7771	

		95% Confidence Interval for Mean	Upper Bound	.9436	
		5% Trimmed Mean		.8304	
		Median		.8200	
		Variance		.048	
		Std. Deviation		.21882	
		Minimum		.63	
		Maximum		1.80	
		Range		1.17	
		Interquartile Range		.17	
		Skewness		3.095	.434
		Kurtosis		12.167	.845
	CHC	Mean		.9067	.06434
		95% Confidence Interval for Mean	Lower Bound	.7687	
			Upper Bound	1.0447	
		5% Trimmed Mean		.8907	
		Median		.8000	
		Variance		.062	
		Std. Deviation		.24919	
		Minimum		.60	
		Maximum		1.50	
		Range		.90	
		Interquartile Range		.40	
		Skewness		1.063	.580
		Kurtosis		.698	1.121
	CHB+C	Mean		1.0100	.05044
		95% Confidence Interval for Mean	Lower Bound	.8959	
			Upper Bound	1.1241	
		5% Trimmed Mean		1.0111	
		Median		1.0000	
		Variance		.025	
		Std. Deviation		.15951	
		Minimum		.80	

	Maximum	1.20	
	Range	.40	
	Interquartile Range	.32	
	Skewness	.004	.687
	Kurtosis	-1.589	1.334

Tests of Normality

		Kolmogorov-Smirnov[a]			Shapiro-Wilk		
	CHB, CHC, CHB+C	Statistic	df	Sig.	Statistic	df	Sig.
AST(U/L)	CHB	.307	29	.000	.451	29	.000
	CHC	.232	15	.028	.785	15	.002
	CHB+C	.140	10	.200*	.960	10	.783
ALT (U/L)	CHB	.165	29	.042	.760	29	.000
	CHC	.276	15	.003	.778	15	.002
	CHB+C	.217	10	.200*	.928	10	.432
GGT (U/L)	CHB	.262	29	.000	.725	29	.000
	CHC	.351	15	.000	.744	15	.001
	CHB+C	.136	10	.200*	.962	10	.809
CHOL (mg/dL)	CHB	.147	29	.113	.975	29	.714
	CHC	.167	15	.200*	.886	15	.059
	CHB+C	.120	10	.200*	.978	10	.952
TG (mg/dL)	CHB	.111	29	.200*	.971	29	.590
	CHC	.086	15	.200*	.958	15	.666
	CHB+C	.254	10	.067	.854	10	.064
ALB (g/dL)	CHB	.204	29	.003	.862	29	.001
	CHC	.207	15	.083	.838	15	.012
	CHB+C	.214	10	.200*	.891	10	.174
PLT	CHB	.129	29	.200*	.984	29	.934
	CHC	.110	15	.200*	.940	15	.382
	CHB+C	.220	10	.185	.936	10	.514
INR	CHB	.168	29	.036	.906	29	.014
	CHC	.119	15	.200*	.963	15	.739
	CHB+C	.265	10	.045	.934	10	.487

TBIL	CHB	.164	29	.045	.876	29	.003
	CHC	.100	15	.200*	.962	15	.720
	CHB+C	.131	10	.200*	.961	10	.801
creatinina serica	CHB	.255	29	.000	.688	29	.000
	CHC	.199	15	.113	.896	15	.082
	CHB+C	.183	10	.200*	.884	10	.145

*. This is a lower bound of the true significance. Lilliefors Significance Correction

Table S3. Principal Component Analysis

KMO and Bartlett's Test

Kaiser-Meyer-Olkin Measure of Sampling Adequacy.		.611
Bartlett's Test of Sphericity	Approx. Chi-Square	98.551
	df	45
	Sig.	.000

Communalities

	Initial
AST(U/L)	1.000
ALT (U/L)	1.000
GGT (U/L)	1.000
CHOL (mg/dL)	1.000
TG (mg/dL)	1.000
ALB (g/dL)	1.000
PLT	1.000
INR	1.000
TBIL	1.000
creatinina serica	1.000

Extraction Method: Principal Component Analysis.

Total Variance Explained

Component	Initial Eigenvalues			Rotation Sums of Squared Loadings		
	Total	% of Variance	Cumulative %	Total	% of Variance	Cumulative %
1	2.665	26.649	26.649	2.343	23.431	23.431
2	1.539	15.395	42.044	1.623	16.231	39.662
3	1.285	12.850	54.894	1.523	15.232	54.894
4	.952	9.524	64.418			
5	.851	8.507	72.925			
6	.763	7.631	80.556			
7	.709	7.090	87.646			
8	.586	5.865	93.511			
9	.409	4.094	97.605			
10	.240	2.395	100.000			

Extraction Method: Principal Component Analysis.

Component Matrix[a]

a. 3 components extracted.

Rotated Component Matrix[a]

	Component		
	1	2	3
AST(U/L)	.804	-.027	.028
ALT (U/L)	.803	.249	.246
GGT (U/L)	.729	-.058	-.078
CHOL (mg/dL)	.002	-.225	.837
TG (mg/dL)	.151	.157	.844
ALB (g/dL)	-.069	.716	-.050
PLT	.460	.300	.039
INR	.491	.064	.066
TBIL	.147	.739	.141
creatinina serica	.132	.573	-.117

Extraction Method: Principal Component Analysis.
Rotation Method: Varimax with Kaiser Normalization.
a. Rotation converged in 5 iterations.

Component Transformation Matrix

Component	1	2	3
1	.875	.396	.279
2	-.084	.692	-.717
3	-.477	.604	.639

Extraction Method: Principal Component Analysis.
Rotation Method: Varimax with Kaiser Normalization.

Table S4. Spearman correlation

Correlations

			TGFb H-score	SMA H-score	GFAP H-score	Fibrosis stage 0-4
Spearman's rho	TGFb H- score	Correlation Coefficient	1.000	.975**	.835**	.756**
		Sig. (2-tailed)	.	.000	.000	.000
		N	54	54	54	54
	SMA H-score	Correlation Coefficient	.975**	1.000	.835**	.774**
		Sig. (2-tailed)	.000	.	.000	.000
		N	54	54	54	54
	GFAP H-score	Correlation Coefficient	.835**	.835**	1.000	.777**
		Sig. (2-tailed)	.000	.000	.	.000

		N	54	54	54	54
	Fibrosis stage 0-4	Correlation Coefficient	.756**	.774**	.777**	1.000
		Sig. (2-tailed)	.000	.000	.000	.
		N	54	54	54	54

**. Correlation is significant at the 0.01 level (2-tailed).

Table S5. Mann–Whitney U test

Ranks

	Fibrosis_group	N	Mean Rank	Sum of Ranks
TGFb H- score	.00	2	1.50	3.00
	1.00	52	28.50	1482.00
	Total	54		
CD5L H-score	.00	2	1.50	3.00
	1.00	52	28.50	1482.00
	Total	54		
SMA H-score	.00	2	1.50	3.00
	1.00	52	28.50	1482.00
	Total	54		
GFAP H-score	.00	2	1.50	3.00
	1.00	52	28.50	1482.00
	Total	54		

Test Statistics[a]

	TGFb H- score	CD5L H-score	SMA H-score	GFAP H-score

Mann-Whitney U	.000	.000	.000	.000
Wilcoxon W	3.000	3.000	3.000	3.000
Z	-2.384	-2.387	-2.387	-2.386
Asymp. Sig. (2-tailed)	.017	.017	.017	.017
Exact Sig. [2*(1-tailed Sig.)]	.001[b]	.001[b]	.001[b]	.001[b]

a. Grouping Variable: Fibrosis_group b. Not corrected for ties

.

Table S6. Fibrosis staging by diagnosis (descriptive statistics – explore)

Case Processing Summary

		Cases					
		Valid		Missing		Total	
	CHB, CHC, CHB+C	N	Percent	N	Percent	N	Percent
Fibrosis stage 0-6	CHB	29	100.0%	0	0.0%	29	100.0%
	CHC	15	100.0%	0	0.0%	15	100.0%
	CHB+C	10	100.0%	0	0.0%	10	100.0%
Fibrosis stage 0-4	CHB	29	100.0%	0	0.0%	29	100.0%
	CHC	15	100.0%	0	0.0%	15	100.0%
	CHB+C	10	100.0%	0	0.0%	10	100.0%
Piece-meal necrosis	CHB	29	100.0%	0	0.0%	29	100.0%
	CHC	15	100.0%	0	0.0%	15	100.0%
	CHB+C	10	100.0%	0	0.0%	10	100.0%
Confluent necrosis	CHB	29	100.0%	0	0.0%	29	100.0%
	CHC	15	100.0%	0	0.0%	15	100.0%
	CHB+C	10	100.0%	0	0.0%	10	100.0%
Focal necrosis/apoptosis	CHB	29	100.0%	0	0.0%	29	100.0%
	CHC	15	100.0%	0	0.0%	15	100.0%
	CHB+C	10	100.0%	0	0.0%	10	100.0%
Portal inflammation	CHB	29	100.0%	0	0.0%	29	100.0%
	CHC	15	100.0%	0	0.0%	15	100.0%
	CHB+C	10	100.0%	0	0.0%	10	100.0%
Total necroinflammatory activity	CHB	29	100.0%	0	0.0%	29	100.0%
	CHC	15	100.0%	0	0.0%	15	100.0%
	CHB+C	10	100.0%	0	0.0%	10	100.0%
METAVIR activity	CHB	29	100.0%	0	0.0%	29	100.0%

	CHC	15	100.0%	0	0.0%	15	100.0%
	CHB+C	10	100.0%	0	0.0%	10	100.0%
Histologic Activity Index	CHB	29	100.0%	0	0.0%	29	100.0%
	CHC	15	100.0%	0	0.0%	15	100.0%
	CHB+C	10	100.0%	0	0.0%	10	100.0%
Portal_necroinflammation_I 0-4	CHB	29	100.0%	0	0.0%	29	100.0%
	CHC	15	100.0%	0	0.0%	15	100.0%
	CHB+C	10	100.0%	0	0.0%	10	100.0%
Portal_necroinflammation_II 0-3	CHB	29	100.0%	0	0.0%	29	100.0%
	CHC	15	100.0%	0	0.0%	15	100.0%
	CHB+C	10	100.0%	0	0.0%	10	100.0%
Portal_necroinflammation_III 0-3	CHB	29	100.0%	0	0.0%	29	100.0%
	CHC	15	100.0%	0	0.0%	15	100.0%
	CHB+C	10	100.0%	0	0.0%	10	100.0%
MELD 3.0	CHB	29	100.0%	0	0.0%	29	100.0%
	CHC	15	100.0%	0	0.0%	15	100.0%
	CHB+C	10	100.0%	0	0.0%	10	100.0%
MELD	CHB	29	100.0%	0	0.0%	29	100.0%
	CHC	15	100.0%	0	0.0%	15	100.0%
	CHB+C	10	100.0%	0	0.0%	10	100.0%

Descriptives

	CHB, CHC, CHB+C			Statistic	Std. Error
Fibrosis stage 0-6	CHB	Mean		4.2414	.27478
		95% Confidence Interval for Mean	Lower Bound	3.6785	
			Upper Bound	4.8042	
		5% Trimmed Mean		4.2682	
		Median		4.0000	
		Variance		2.190	
		Std. Deviation		1.47975	
		Minimum		2.00	
		Maximum		6.00	
		Range		4.00	
		Interquartile Range		3.00	
		Skewness		.192	.434
		Kurtosis		-1.672	.845
	CHC	Mean		4.0667	.39601
			Lower Bound	3.2173	

		95% Confidence Interval for Mean	Upper Bound	4.9160	
		5% Trimmed Mean		4.0741	
		Median		3.0000	
		Variance		2.352	
		Std. Deviation		1.53375	
		Minimum		2.00	
		Maximum		6.00	
		Range		4.00	
		Interquartile Range		3.00	
		Skewness		.146	.580
		Kurtosis		-1.735	1.121
	CHB+C	Mean		3.7000	.51747
		95% Confidence Interval for Mean	Lower Bound	2.5294	
			Upper Bound	4.8706	
		5% Trimmed Mean		3.6667	
		Median		3.0000	
		Variance		2.678	
		Std. Deviation		1.63639	
		Minimum		2.00	
		Maximum		6.00	
		Range		4.00	
		Interquartile Range		3.25	
		Skewness		.791	.687
		Kurtosis		-1.239	1.334
Fibrosis stage 0-4	CHB	Mean		2.7586	.20254
		95% Confidence Interval for Mean	Lower Bound	2.3437	
			Upper Bound	3.1735	
		5% Trimmed Mean		2.7874	
		Median		2.0000	
		Variance		1.190	
		Std. Deviation		1.09071	
		Minimum		1.00	

				Statistic	Std. Error
		Maximum		4.00	
		Range		3.00	
		Interquartile Range		2.00	
		Skewness		.163	.434
		Kurtosis		-1.706	.845
	CHC	Mean		3.3333	.18687
		95% Confidence Interval for Mean	Lower Bound	2.9325	
			Upper Bound	3.7341	
		5% Trimmed Mean		3.3704	
		Median		3.0000	
		Variance		.524	
		Std. Deviation		.72375	
		Minimum		2.00	
		Maximum		4.00	
		Range		2.00	
		Interquartile Range		1.00	
		Skewness		-.628	.580
		Kurtosis		-.654	1.121
	CHB+C	Mean		2.7000	.21344
		95% Confidence Interval for Mean	Lower Bound	2.2172	
			Upper Bound	3.1828	
		5% Trimmed Mean		2.6667	
		Median		3.0000	
		Variance		.456	
		Std. Deviation		.67495	
		Minimum		2.00	
		Maximum		4.00	
		Range		2.00	
		Interquartile Range		1.00	
		Skewness		.434	.687
		Kurtosis		-.283	1.334
Piece-meal necrosis	CHB	Mean		2.8276	.13195
			Lower Bound	2.5573	

		95% Confidence Interval for Mean	Upper Bound	3.0979	
		5% Trimmed Mean		2.8467	
		Median		3.0000	
		Variance		.505	
		Std. Deviation		.71058	
		Minimum		1.00	
		Maximum		4.00	
		Range		3.00	
		Interquartile Range		1.00	
		Skewness		-.378	.434
		Kurtosis		.471	.845
	CHC	Mean		2.6667	.12599
		95% Confidence Interval for Mean	Lower Bound	2.3964	
			Upper Bound	2.9369	
		5% Trimmed Mean		2.6852	
		Median		3.0000	
		Variance		.238	
		Std. Deviation		.48795	
		Minimum		2.00	
		Maximum		3.00	
		Range		1.00	
		Interquartile Range		1.00	
		Skewness		-.788	.580
		Kurtosis		-1.615	1.121
	CHB+C	Mean		3.0000	.14907
		95% Confidence Interval for Mean	Lower Bound	2.6628	
			Upper Bound	3.3372	
		5% Trimmed Mean		3.0000	
		Median		3.0000	
		Variance		.222	
		Std. Deviation		.47140	
		Minimum		2.00	

		Maximum		4.00	
		Range		2.00	
		Interquartile Range		.00	
		Skewness		.000	.687
		Kurtosis		4.500	1.334
Confluent necrosis	CHB	Mean		.0000	.00000
		95% Confidence Interval for Mean	Lower Bound	.0000	
			Upper Bound	.0000	
		5% Trimmed Mean		.0000	
		Median		.0000	
		Variance		.000	
		Std. Deviation		.00000	
		Minimum		.00	
		Maximum		.00	
		Range		.00	
		Interquartile Range		.00	
		Skewness		.	.
		Kurtosis		.	.
	CHC	Mean		.0000	.00000
		95% Confidence Interval for Mean	Lower Bound	.0000	
			Upper Bound	.0000	
		5% Trimmed Mean		.0000	
		Median		.0000	
		Variance		.000	
		Std. Deviation		.00000	
		Minimum		.00	
		Maximum		.00	
		Range		.00	
		Interquartile Range		.00	
		Skewness		.	.
		Kurtosis		.	.
	CHB+C	Mean		.0000	.00000
			Lower Bound	.0000	

		95% Confidence Interval for Mean	Upper Bound	.0000	
		5% Trimmed Mean		.0000	
		Median		.0000	
		Variance		.000	
		Std. Deviation		.00000	
		Minimum		.00	
		Maximum		.00	
		Range		.00	
		Interquartile Range		.00	
		Skewness		.	.
		Kurtosis		.	.
Focal necrosis/apoptosis	CHB	Mean		2.3448	.10262
		95% Confidence Interval for Mean	Lower Bound	2.1346	
			Upper Bound	2.5550	
		5% Trimmed Mean		2.3659	
		Median		2.0000	
		Variance		.305	
		Std. Deviation		.55265	
		Minimum		1.00	
		Maximum		3.00	
		Range		2.00	
		Interquartile Range		1.00	
		Skewness		-.008	.434
		Kurtosis		-.723	.845
	CHC	Mean		2.0000	.00000
		95% Confidence Interval for Mean	Lower Bound	2.0000	
			Upper Bound	2.0000	
		5% Trimmed Mean		2.0000	
		Median		2.0000	
		Variance		.000	
		Std. Deviation		.00000	
		Minimum		2.00	

		Maximum		2.00	
		Range		.00	
		Interquartile Range		.00	
		Skewness		.	.
		Kurtosis		.	.
	CHB+C	Mean		2.2000	.13333
		95% Confidence Interval for Mean	Lower Bound	1.8984	
			Upper Bound	2.5016	
		5% Trimmed Mean		2.1667	
		Median		2.0000	
		Variance		.178	
		Std. Deviation		.42164	
		Minimum		2.00	
		Maximum		3.00	
		Range		1.00	
		Interquartile Range		.25	
		Skewness		1.779	.687
		Kurtosis		1.406	1.334
Portal inflammation	CHB	Mean		2.4828	.10668
		95% Confidence Interval for Mean	Lower Bound	2.2642	
			Upper Bound	2.7013	
		5% Trimmed Mean		2.5192	
		Median		3.0000	
		Variance		.330	
		Std. Deviation		.57450	
		Minimum		1.00	
		Maximum		3.00	
		Range		2.00	
		Interquartile Range		1.00	
		Skewness		-.535	.434
		Kurtosis		-.663	.845
	CHC	Mean		2.4000	.13093
			Lower Bound	2.1192	

		95% Confidence Interval for Mean	Upper Bound	2.6808	
		5% Trimmed Mean		2.3889	
		Median		2.0000	
		Variance		.257	
		Std. Deviation		.50709	
		Minimum		2.00	
		Maximum		3.00	
		Range		1.00	
		Interquartile Range		1.00	
		Skewness		.455	.580
		Kurtosis		-2.094	1.121
	CHB+C	Mean		2.6000	.16330
		95% Confidence Interval for Mean	Lower Bound	2.2306	
			Upper Bound	2.9694	
		5% Trimmed Mean		2.6111	
		Median		3.0000	
		Variance		.267	
		Std. Deviation		.51640	
		Minimum		2.00	
		Maximum		3.00	
		Range		1.00	
		Interquartile Range		1.00	
		Skewness		-.484	.687
		Kurtosis		-2.277	1.334
Total necroinflammatory activity	CHB	Mean		7.6552	.29476
		95% Confidence Interval for Mean	Lower Bound	7.0514	
			Upper Bound	8.2590	
		5% Trimmed Mean		7.7874	
		Median		9.0000	
		Variance		2.520	
		Std. Deviation		1.58736	
		Minimum		3.00	

		Maximum		9.00	
		Range		6.00	
		Interquartile Range		3.00	
		Skewness		-.938	.434
		Kurtosis		.640	.845
	CHC	Mean		7.0667	.20625
		95% Confidence Interval for Mean	Lower Bound	6.6243	
			Upper Bound	7.5090	
		5% Trimmed Mean		7.0741	
		Median		7.0000	
		Variance		.638	
		Std. Deviation		.79881	
		Minimum		6.00	
		Maximum		8.00	
		Range		2.00	
		Interquartile Range		2.00	
		Skewness		-.128	.580
		Kurtosis		-1.348	1.121
	CHB+C	Mean		7.8000	.35901
		95% Confidence Interval for Mean	Lower Bound	6.9879	
			Upper Bound	8.6121	
		5% Trimmed Mean		7.7778	
		Median		8.0000	
		Variance		1.289	
		Std. Deviation		1.13529	
		Minimum		6.00	
		Maximum		10.00	
		Range		4.00	
		Interquartile Range		1.25	
		Skewness		.478	.687
		Kurtosis		.552	1.334
METAVIR activity	CHB	Mean		1.4828	.10668
			Lower Bound	1.2642	

		95% Confidence Interval for Mean	Upper Bound	1.7013	
		5% Trimmed Mean		1.5192	
		Median		2.0000	
		Variance		.330	
		Std. Deviation		.57450	
		Minimum		.00	
		Maximum		2.00	
		Range		2.00	
		Interquartile Range		1.00	
		Skewness		-.535	.434
		Kurtosis		-.663	.845
	CHC	Mean		2.0000	.00000
		95% Confidence Interval for Mean	Lower Bound	2.0000	
			Upper Bound	2.0000	
		5% Trimmed Mean		2.0000	
		Median		2.0000	
		Variance		.000	
		Std. Deviation		.00000	
		Minimum		2.00	
		Maximum		2.00	
		Range		.00	
		Interquartile Range		.00	
		Skewness		.	.
		Kurtosis		.	.
	CHB+C	Mean		2.0000	.00000
		95% Confidence Interval for Mean	Lower Bound	2.0000	
			Upper Bound	2.0000	
		5% Trimmed Mean		2.0000	
		Median		2.0000	
		Variance		.000	
		Std. Deviation		.00000	
		Minimum		2.00	

		Maximum		2.00	
		Range		.00	
		Interquartile Range		.00	
		Skewness		.	.
		Kurtosis		.	.
Histologic Activity Index	CHB	Mean		7.6552	.31496
		95% Confidence Interval for Mean	Lower Bound	7.0100	
			Upper Bound	8.3003	
		5% Trimmed Mean		7.7663	
		Median		9.0000	
		Variance		2.877	
		Std. Deviation		1.69613	
		Minimum		3.00	
		Maximum		10.00	
		Range		7.00	
		Interquartile Range		2.50	
		Skewness		-.876	.434
		Kurtosis		.267	.845
	CHC	Mean		7.2000	.34087
		95% Confidence Interval for Mean	Lower Bound	6.4689	
			Upper Bound	7.9311	
		5% Trimmed Mean		7.1667	
		Median		7.0000	
		Variance		1.743	
		Std. Deviation		1.32017	
		Minimum		5.00	
		Maximum		10.00	
		Range		5.00	
		Interquartile Range		1.00	
		Skewness		.224	.580
		Kurtosis		.649	1.121
	CHB+C	Mean		7.6000	.26667
			Lower Bound	6.9968	

		95% Confidence Interval for Mean	Upper Bound	8.2032	
		5% Trimmed Mean		7.6111	
		Median		8.0000	
		Variance		.711	
		Std. Deviation		.84327	
		Minimum		6.00	
		Maximum		9.00	
		Range		3.00	
		Interquartile Range		1.00	
		Skewness		-.389	.687
		Kurtosis		.370	1.334
Portal_necroinflammation_I 0-4	CHB	Mean		2.6897	.13227
		95% Confidence Interval for Mean	Lower Bound	2.4187	
			Upper Bound	2.9606	
		5% Trimmed Mean		2.7280	
		Median		3.0000	
		Variance		.507	
		Std. Deviation		.71231	
		Minimum		1.00	
		Maximum		4.00	
		Range		3.00	
		Interquartile Range		.50	
		Skewness		-1.370	.434
		Kurtosis		1.549	.845
	CHC	Mean		2.6000	.23503
		95% Confidence Interval for Mean	Lower Bound	2.0959	
			Upper Bound	3.1041	
		5% Trimmed Mean		2.6111	
		Median		3.0000	
		Variance		.829	
		Std. Deviation		.91026	
		Minimum		1.00	

		Maximum		4.00	
		Range		3.00	
		Interquartile Range		1.00	
		Skewness		-.997	.580
		Kurtosis		.054	1.121
	CHB+C	Mean		3.1000	.17951
		95% Confidence Interval for Mean	Lower Bound	2.6939	
			Upper Bound	3.5061	
		5% Trimmed Mean		3.1111	
		Median		3.0000	
		Variance		.322	
		Std. Deviation		.56765	
		Minimum		2.00	
		Maximum		4.00	
		Range		2.00	
		Interquartile Range		.25	
		Skewness		.091	.687
		Kurtosis		1.498	1.334
Portal_necroinflammation_II 0-3	CHB	Mean		2.4138	.13607
		95% Confidence Interval for Mean	Lower Bound	2.1351	
			Upper Bound	2.6925	
		5% Trimmed Mean		2.4598	
		Median		3.0000	
		Variance		.537	
		Std. Deviation		.73277	
		Minimum		1.00	
		Maximum		3.00	
		Range		2.00	
		Interquartile Range		1.00	
		Skewness		-.850	.434
		Kurtosis		-.566	.845
	CHC	Mean		1.6667	.21082
			Lower Bound	1.2145	

		95% Confidence Interval for Mean	Upper Bound	2.1188	
		5% Trimmed Mean		1.6296	
		Median		1.0000	
		Variance		.667	
		Std. Deviation		.81650	
		Minimum		1.00	
		Maximum		3.00	
		Range		2.00	
		Interquartile Range		1.00	
		Skewness		.740	.580
		Kurtosis		-1.022	1.121
	CHB+C	Mean		1.8000	.20000
		95% Confidence Interval for Mean	Lower Bound	1.3476	
			Upper Bound	2.2524	
		5% Trimmed Mean		1.7778	
		Median		2.0000	
		Variance		.400	
		Std. Deviation		.63246	
		Minimum		1.00	
		Maximum		3.00	
		Range		2.00	
		Interquartile Range		1.00	
		Skewness		.132	.687
		Kurtosis		.179	1.334
Portal_necroinflammation_III 0-3	CHB	Mean		2.5517	.11730
		95% Confidence Interval for Mean	Lower Bound	2.3114	
			Upper Bound	2.7920	
		5% Trimmed Mean		2.6130	
		Median		3.0000	
		Variance		.399	
		Std. Deviation		.63168	
		Minimum		1.00	

		Maximum		3.00	
		Range		2.00	
		Interquartile Range		1.00	
		Skewness		-1.121	.434
		Kurtosis		.307	.845
	CHC	Mean		2.9333	.06667
		95% Confidence Interval for Mean	Lower Bound	2.7903	
			Upper Bound	3.0763	
		5% Trimmed Mean		2.9815	
		Median		3.0000	
		Variance		.067	
		Std. Deviation		.25820	
		Minimum		2.00	
		Maximum		3.00	
		Range		1.00	
		Interquartile Range		.00	
		Skewness		-3.873	.580
		Kurtosis		15.000	1.121
	CHB+C	Mean		2.7000	.15275
		95% Confidence Interval for Mean	Lower Bound	2.3544	
			Upper Bound	3.0456	
		5% Trimmed Mean		2.7222	
		Median		3.0000	
		Variance		.233	
		Std. Deviation		.48305	
		Minimum		2.00	
		Maximum		3.00	
		Range		1.00	
		Interquartile Range		1.00	
		Skewness		-1.035	.687
		Kurtosis		-1.224	1.334
MELD 3.0	CHB	Mean		19.0345	.29143
			Lower Bound	18.4375	

		95% Confidence Interval for Mean	Upper Bound	19.6315	
		5% Trimmed Mean		18.9617	
		Median		19.0000	
		Variance		2.463	
		Std. Deviation		1.56941	
		Minimum		16.00	
		Maximum		24.00	
		Range		8.00	
		Interquartile Range		2.00	
		Skewness		.951	.434
		Kurtosis		2.393	.845
	CHC	Mean		18.6667	.42164
		95% Confidence Interval for Mean	Lower Bound	17.7623	
			Upper Bound	19.5710	
		5% Trimmed Mean		18.5741	
		Median		19.0000	
		Variance		2.667	
		Std. Deviation		1.63299	
		Minimum		16.00	
		Maximum		23.00	
		Range		7.00	
		Interquartile Range		1.00	
		Skewness		1.077	.580
		Kurtosis		2.810	1.121
	CHB+C	Mean		18.8000	.62893
		95% Confidence Interval for Mean	Lower Bound	17.3773	
			Upper Bound	20.2227	
		5% Trimmed Mean		18.7222	
		Median		18.0000	
		Variance		3.956	
		Std. Deviation		1.98886	
		Minimum		17.00	

		Maximum		22.00	
		Range		5.00	
		Interquartile Range		4.00	
		Skewness		.555	.687
		Kurtosis		-1.597	1.334
MELD	CHB	Mean		7.4828	.31239
		95% Confidence Interval for Mean	Lower Bound	6.8429	
			Upper Bound	8.1227	
		5% Trimmed Mean		7.2720	
		Median		7.0000	
		Variance		2.830	
		Std. Deviation		1.68228	
		Minimum		6.00	
		Maximum		14.00	
		Range		8.00	
		Interquartile Range		1.50	
		Skewness		2.352	.434
		Kurtosis		7.314	.845
	CHC	Mean		7.0667	.28396
		95% Confidence Interval for Mean	Lower Bound	6.4576	
			Upper Bound	7.6757	
		5% Trimmed Mean		6.9630	
		Median		7.0000	
		Variance		1.210	
		Std. Deviation		1.09978	
		Minimum		6.00	
		Maximum		10.00	
		Range		4.00	
		Interquartile Range		2.00	
		Skewness		1.339	.580
		Kurtosis		2.449	1.121
	CHB+C	Mean		9.3000	.49554
			Lower Bound	8.1790	

		95% Confidence Interval for Mean	Upper Bound	10.4210	
		5% Trimmed Mean		9.2778	
		Median		9.0000	
		Variance		2.456	
		Std. Deviation		1.56702	
		Minimum		7.00	
		Maximum		12.00	
		Range		5.00	
		Interquartile Range		3.00	
		Skewness		.463	.687
		Kurtosis		-.590	1.334

Tests of Normality

		Kolmogorov-Smirnov[a]			Shapiro-Wilk		
	CHB, CHC, CHB+C	Statistic	df	Sig.	Statistic	df	Sig.
Fibrosis stage 0-6	CHB	.262	29	.000	.790	29	.000
	CHC	.290	15	.001	.830	15	.009
	CHB+C	.366	10	.000	.751	10	.004
Fibrosis stage 0-4	CHB	.343	29	.000	.728	29	.000
	CHC	.288	15	.002	.783	15	.002
	CHB+C	.272	10	.035	.802	10	.015
Piece-meal necrosis	CHB	.320	29	.000	.825	29	.000
	CHC	.419	15	.000	.603	15	.000
	CHB+C	.400	10	.000	.658	10	.000
Confluent necrosis	CHB	.	29	.	.	29	.
	CHC	.	15	.	.	15	.
	CHB+C	.	10	.	.	10	.
Focal necrosis/apoptosis	CHB	.354	29	.000	.716	29	.000
	CHC	.	15	.	.	15	.
	CHB+C	.482	10	.000	.509	10	.000
Portal inflammation	CHB	.333	29	.000	.715	29	.000
	CHC	.385	15	.000	.630	15	.000
	CHB+C	.381	10	.000	.640	10	.000
Total necroinflammatory activity	CHB	.319	29	.000	.769	29	.000
	CHC	.212	15	.068	.817	15	.006
	CHB+C	.230	10	.143	.933	10	.479
METAVIR activity	CHB	.333	29	.000	.715	29	.000
	CHC	.	15	.	.	15	.

	CHB+C	.	10	.	.	10	.
Histologic Activity Index	CHB	.303	29	.000	.839	29	.000
	CHC	.240	15	.020	.913	15	.150
	CHB+C	.282	10	.023	.890	10	.172
Portal_necroinflammation_I 0-4	CHB	.427	29	.000	.667	29	.000
	CHC	.403	15	.000	.721	15	.000
	CHB+C	.370	10	.000	.752	10	.004
Portal_necroinflammation_II 0-3	CHB	.340	29	.000	.735	29	.000
	CHC	.326	15	.000	.749	15	.001
	CHB+C	.324	10	.004	.794	10	.012
Portal_necroinflammation_III 0-3	CHB	.382	29	.000	.690	29	.000
	CHC	.535	15	.000	.284	15	.000
	CHB+C	.433	10	.000	.594	10	.000
MELD 3.0	CHB	.164	29	.045	.914	29	.022
	CHC	.219	15	.051	.901	15	.098
	CHB+C	.256	10	.062	.822	10	.027
MELD	CHB	.303	29	.000	.734	29	.000
	CHC	.258	15	.008	.822	15	.007
	CHB+C	.276	10	.030	.916	10	.322

a. Lilliefors Significance Correction

Table S7. Ishak necroinflammatory scores system - Sex-Based Analysis

Descriptive Statistics

	N	Mean	Std. Deviation	Minimum	Maximum
Piece-meal necrosis	54	2.8148	.61657	1.00	4.00
Confluent necrosis	54	.0000	.00000	.00	.00
Focal necrosis/apoptosis	54	2.2222	.46242	1.00	3.00
Portal inflammation	54	2.4815	.54047	1.00	3.00
Total necroinflammatory activity	54	7.5185	1.34221	3.00	10.00
METAVIR activity	54	1.7222	.49208	.00	2.00
male, female	54	.5370	.50331	.00	1.00

Ranks

	male, female	N	Mean Rank	Sum of Ranks
Piece-meal necrosis	female	25	27.24	681.00
	male	29	27.72	804.00
	Total	54		
Confluent necrosis	female	25	27.50	687.50

	male	29	27.50	797.50
	Total	54		
Focal necrosis/apoptosis	female	25	30.22	755.50
	male	29	25.16	729.50
	Total	54		
Portal inflammation	female	25	27.74	693.50
	male	29	27.29	791.50
	Total	54		
Total necroinflammatory activity	female	25	29.00	725.00
	male	29	26.21	760.00
	Total	54		
METAVIR activity	female	25	25.74	643.50
	male	29	29.02	841.50
	Total	54		

Test Statistics[a]

	Piece-meal necrosis	Confluent necrosis	Focal necrosis/apoptosis	Portal inflammation	Total necroinflammatory activity	METAVIR activity
Mann-Whitney U	356.000	362.500	294.500	356.500	325.000	318.500
Wilcoxon W	681.000	797.500	729.500	791.500	760.000	643.500
Z	-.134	.000	-1.549	-.119	-.672	-1.002
Asymp. Sig. (2-tailed)	.894	1.000	.121	.905	.502	.316

a. Grouping Variable: male, female

Table S8. Ishak necroinflammatory scores system - Age-Based Correlation Analysis

Correlations

			Piece-meal necrosis	Confluent necrosis	Focal necrosis/apoptosis	Portal inflammation	Total necroinflammatory activity	METAVIR activity	Age
Spearman's rho	Piece-meal necrosis	Correlation Coefficient	1.000	.	.311*	.578**	.812**	.449**	-.007
		Sig. (2-tailed)	.	.	.022	.000	.000	.001	.959
		N	54	54	54	54	54	54	54
	Confluent necrosis	Correlation Coefficient	.	.	.	.	.	.	.
		Sig. (2-tailed)	.	.	.	.	.	.	.
		N	54	54	54	54	54	54	54

	Focal necrosis/apoptosis	Correlation Coefficient	.311*	.	1.000	.595**	.721**	.389**	.420**
		Sig. (2-tailed)	.022	.	.	.000	.000	.004	.002
		N	54	54	54	54	54	54	54
	Portal inflammation	Correlation Coefficient	.578**	.	.595**	1.000	.886**	.615**	.156
		Sig. (2-tailed)	.000	.	.000	.	.000	.000	.259
		N	54	54	54	54	54	54	54
	Total necroinflammatory activity	Correlation Coefficient	.812**	.	.721**	.886**	1.000	.582**	.196
		Sig. (2-tailed)	.000	.	.000	.000	.	.000	.155
		N	54	54	54	54	54	54	54
	METAVIR activity	Correlation Coefficient	.449**	.	.389**	.615**	.582**	1.000	.119
		Sig. (2-tailed)	.001	.	.004	.000	.000	.	.390
		N	54	54	54	54	54	54	54
	Age	Correlation Coefficient	-.007	.	.420**	.156	.196	.119	1.000
		Sig. (2-tailed)	.959	.	.002	.259	.155	.390	.
		N	54	54	54	54	54	54	54

*. Correlation is significant at the 0.05 level (2-tailed). **. Correlation is significant at the 0.01 level (2-tailed).

Table S9. H-score (descriptive statistics – explore)

Case Processing Summary

		Cases					
		Valid		Missing		Total	
	CHB, CHC, CHB+C	N	Percent	N	Percent	N	Percent
TGFb H- score	CHB	29	100.0%	0	0.0%	29	100.0%
	CHC	15	100.0%	0	0.0%	15	100.0%
	CHB+C	10	100.0%	0	0.0%	10	100.0%
CD5L H-score	CHB	29	100.0%	0	0.0%	29	100.0%
	CHC	15	100.0%	0	0.0%	15	100.0%
	CHB+C	10	100.0%	0	0.0%	10	100.0%
SMA H-score	CHB	29	100.0%	0	0.0%	29	100.0%
	CHC	15	100.0%	0	0.0%	15	100.0%
	CHB+C	10	100.0%	0	0.0%	10	100.0%
GFAP H-score	CHB	29	100.0%	0	0.0%	29	100.0%
	CHC	15	100.0%	0	0.0%	15	100.0%

	CHB+C	10	100.0%	0	0.0%	10	100.0%
Fibrosis stage 0-6	CHB	29	100.0%	0	0.0%	29	100.0%
	CHC	15	100.0%	0	0.0%	15	100.0%
	CHB+C	10	100.0%	0	0.0%	10	100.0%
Fibrosis stage 0-4	CHB	29	100.0%	0	0.0%	29	100.0%
	CHC	15	100.0%	0	0.0%	15	100.0%
	CHB+C	10	100.0%	0	0.0%	10	100.0%

Descriptives

	CHB, CHC, CHB+C			Statistic	Std. Error
TGFb H- score	CHB	Mean		162.4483	9.96775
		95% Confidence Interval for Mean	Lower Bound	142.0303	
			Upper Bound	182.8663	
		5% Trimmed Mean		163.8429	
		Median		136.0000	
		Variance		2881.328	
		Std. Deviation		53.67800	
		Minimum		65.00	
		Maximum		232.00	
		Range		167.00	
		Interquartile Range		101.00	
		Skewness		.082	.434
		Kurtosis		-1.415	.845
	CHC	Mean		189.3333	10.45246
		95% Confidence Interval for Mean	Lower Bound	166.9150	
			Upper Bound	211.7516	
		5% Trimmed Mean		190.3704	
		Median		175.0000	
		Variance		1638.810	
		Std. Deviation		40.48221	
		Minimum		120.00	
		Maximum		240.00	
		Range		120.00	

		Interquartile Range		55.00	
		Skewness		-.385	.580
		Kurtosis		-1.051	1.121
	CHB+C	Mean		190.5000	11.70114
		95% Confidence Interval for Mean	Lower Bound	164.0302	
			Upper Bound	216.9698	
		5% Trimmed Mean		190.8333	
		Median		185.0000	
		Variance		1369.167	
		Std. Deviation		37.00225	
		Minimum		135.00	
		Maximum		240.00	
		Range		105.00	
		Interquartile Range		62.50	
		Skewness		.007	.687
		Kurtosis		-.794	1.334
CD5L H-score	CHB	Mean		148.2759	8.61862
		95% Confidence Interval for Mean	Lower Bound	130.6214	
			Upper Bound	165.9303	
		5% Trimmed Mean		150.3257	
		Median		135.0000	
		Variance		2154.135	
		Std. Deviation		46.41266	
		Minimum		50.00	
		Maximum		205.00	
		Range		155.00	
		Interquartile Range		87.50	
		Skewness		-.196	.434
		Kurtosis		-.974	.845
	CHC	Mean		181.6667	11.49189
		95% Confidence Interval for Mean	Lower Bound	157.0190	
			Upper Bound	206.3143	
		5% Trimmed Mean		183.5185	

		Median		175.0000	
		Variance		1980.952	
		Std. Deviation		44.50789	
		Minimum		100.00	
		Maximum		230.00	
		Range		130.00	
		Interquartile Range		60.00	
		Skewness		-.545	.580
		Kurtosis		-.963	1.121
	CHB+C	Mean		198.0000	8.34000
		95% Confidence Interval for Mean	Lower Bound	179.1336	
			Upper Bound	216.8664	
		5% Trimmed Mean		198.3333	
		Median		197.5000	
		Variance		695.556	
		Std. Deviation		26.37339	
		Minimum		155.00	
		Maximum		235.00	
		Range		80.00	
		Interquartile Range		40.00	
		Skewness		-.309	.687
		Kurtosis		-.430	1.334
SMA H-score	CHB	Mean		179.9655	10.60492
		95% Confidence Interval for Mean	Lower Bound	158.2423	
			Upper Bound	201.6887	
		5% Trimmed Mean		182.9119	
		Median		156.0000	
		Variance		3261.463	
		Std. Deviation		57.10922	
		Minimum		55.00	
		Maximum		246.00	
		Range		191.00	
		Interquartile Range		108.00	

		Skewness		-.209	.434
		Kurtosis		-.918	.845
	CHC	Mean		207.6667	11.18956
		95% Confidence Interval for Mean	Lower Bound	183.6674	
			Upper Bound	231.6659	
		5% Trimmed Mean		210.4630	
		Median		215.0000	
		Variance		1878.095	
		Std. Deviation		43.33700	
		Minimum		115.00	
		Maximum		250.00	
		Range		135.00	
		Interquartile Range		40.00	
		Skewness		-1.364	.580
		Kurtosis		1.189	1.121
	CHB+C	Mean		217.5000	10.70436
		95% Confidence Interval for Mean	Lower Bound	193.2851	
			Upper Bound	241.7149	
		5% Trimmed Mean		218.8889	
		Median		220.0000	
		Variance		1145.833	
		Std. Deviation		33.85016	
		Minimum		155.00	
		Maximum		255.00	
		Range		100.00	
		Interquartile Range		45.00	
		Skewness		-.939	.687
		Kurtosis		.213	1.334
GFAP H-score	CHB	Mean		146.4138	8.72648
		95% Confidence Interval for Mean	Lower Bound	128.5384	
			Upper Bound	164.2892	
		5% Trimmed Mean		148.3774	
		Median		130.0000	

		Variance		2208.394	
		Std. Deviation		46.99355	
		Minimum		45.00	
		Maximum		210.00	
		Range		165.00	
		Interquartile Range		86.50	
		Skewness		-.188	.434
		Kurtosis		-.703	.845
	CHC	Mean		151.3333	4.43113
		95% Confidence Interval for Mean	Lower Bound	141.8295	
			Upper Bound	160.8372	
		5% Trimmed Mean		151.7593	
		Median		155.0000	
		Variance		294.524	
		Std. Deviation		17.16170	
		Minimum		120.00	
		Maximum		175.00	
		Range		55.00	
		Interquartile Range		20.00	
		Skewness		-.934	.580
		Kurtosis		-.162	1.121
	CHB+C	Mean		162.0000	3.09121
		95% Confidence Interval for Mean	Lower Bound	155.0072	
			Upper Bound	168.9928	
		5% Trimmed Mean		161.9444	
		Median		160.0000	
		Variance		95.556	
		Std. Deviation		9.77525	
		Minimum		145.00	
		Maximum		180.00	
		Range		35.00	
		Interquartile Range		15.00	
		Skewness		.187	.687

		Kurtosis		.480	1.334
Fibrosis stage 0-6	CHB	Mean		4.2414	.27478
		95% Confidence Interval for Mean	Lower Bound	3.6785	
			Upper Bound	4.8042	
		5% Trimmed Mean		4.2682	
		Median		4.0000	
		Variance		2.190	
		Std. Deviation		1.47975	
		Minimum		2.00	
		Maximum		6.00	
		Range		4.00	
		Interquartile Range		3.00	
		Skewness		.192	.434
		Kurtosis		-1.672	.845
	CHC	Mean		4.0667	.39601
		95% Confidence Interval for Mean	Lower Bound	3.2173	
			Upper Bound	4.9160	
		5% Trimmed Mean		4.0741	
		Median		3.0000	
		Variance		2.352	
		Std. Deviation		1.53375	
		Minimum		2.00	
		Maximum		6.00	
		Range		4.00	
		Interquartile Range		3.00	
		Skewness		.146	.580
		Kurtosis		-1.735	1.121
	CHB+C	Mean		3.7000	.51747
		95% Confidence Interval for Mean	Lower Bound	2.5294	
			Upper Bound	4.8706	
		5% Trimmed Mean		3.6667	
		Median		3.0000	
		Variance		2.678	

		Std. Deviation		1.63639	
		Minimum		2.00	
		Maximum		6.00	
		Range		4.00	
		Interquartile Range		3.25	
		Skewness		.791	.687
		Kurtosis		-1.239	1.334
Fibrosis stage 0-4	CHB	Mean		2.7586	.20254
		95% Confidence Interval for Mean	Lower Bound	2.3437	
			Upper Bound	3.1735	
		5% Trimmed Mean		2.7874	
		Median		2.0000	
		Variance		1.190	
		Std. Deviation		1.09071	
		Minimum		1.00	
		Maximum		4.00	
		Range		3.00	
		Interquartile Range		2.00	
		Skewness		.163	.434
		Kurtosis		-1.706	.845
	CHC	Mean		3.3333	.18687
		95% Confidence Interval for Mean	Lower Bound	2.9325	
			Upper Bound	3.7341	
		5% Trimmed Mean		3.3704	
		Median		3.0000	
		Variance		.524	
		Std. Deviation		.72375	
		Minimum		2.00	
		Maximum		4.00	
		Range		2.00	
		Interquartile Range		1.00	
		Skewness		-.628	.580
		Kurtosis		-.654	1.121

	CHB+C	Mean		2.7000	.21344
		95% Confidence Interval for Mean	Lower Bound	2.2172	
			Upper Bound	3.1828	
		5% Trimmed Mean		2.6667	
		Median		3.0000	
		Variance		.456	
		Std. Deviation		.67495	
		Minimum		2.00	
		Maximum		4.00	
		Range		2.00	
		Interquartile Range		1.00	
		Skewness		.434	.687
		Kurtosis		-.283	1.334

Tests of Normality

		Kolmogorov-Smirnov[a]			Shapiro-Wilk		
	CHB, CHC, CHB+C	Statistic	df	Sig.	Statistic	df	Sig.
TGFb H- score	CHB	.221	29	.001	.865	29	.002
	CHC	.204	15	.095	.887	15	.061
	CHB+C	.259	10	.056	.875	10	.115
CD5L H-score	CHB	.195	29	.006	.891	29	.006
	CHC	.205	15	.088	.883	15	.053
	CHB+C	.181	10	.200*	.927	10	.417
SMA H-score	CHB	.233	29	.000	.855	29	.001
	CHC	.230	15	.032	.824	15	.008
	CHB+C	.271	10	.037	.857	10	.071
GFAP H-score	CHB	.155	29	.074	.913	29	.020
	CHC	.251	15	.012	.862	15	.026
	CHB+C	.181	10	.200*	.967	10	.866
Fibrosis stage 0-6	CHB	.262	29	.000	.790	29	.000
	CHC	.290	15	.001	.830	15	.009
	CHB+C	.366	10	.000	.751	10	.004
Fibrosis stage 0-4	CHB	.343	29	.000	.728	29	.000
	CHC	.288	15	.002	.783	15	.002
	CHB+C	.272	10	.035	.802	10	.015

*. This is a lower bound of the true significance. a. Lilliefors Significance Correction

Table S10. Kruskal-Wallis Test

Ranks

	CHB, CHC, CHB+C	N	Mean Rank
Fibrosis stage 0-6	CHB	29	29.48
	CHC	15	26.60
	CHB+C	10	23.10
	Total	54	
TGFb H- score	CHB	29	23.24
	CHC	15	31.30
	CHB+C	10	34.15
	Total	54	
CD5L H-score	CHB	29	21.50
	CHC	15	33.03
	CHB+C	10	36.60
	Total	54	
SMA H-score	CHB	29	23.90
	CHC	15	29.93
	CHB+C	10	34.30
	Total	54	
GFAP H-score	CHB	29	25.41
	CHC	15	27.40
	CHB+C	10	33.70
	Total	54	
AST(U/L)	CHB	29	22.81
	CHC	15	28.20
	CHB+C	10	40.05
	Total	54	
ALT (U/L)	CHB	29	26.19
	CHC	15	20.00
	CHB+C	10	42.55
	Total	54	
GGT (U/L)	CHB	29	19.69
	CHC	15	33.23
	CHB+C	10	41.55
	Total	54	

Test Statistics[a,b]

	Fibrosis stage 0-6	TGFb H- score	CD5L H-score	SMA H-score	GFAP H-score	AST(U/L)	ALT (U/L)	GGT (U/L)
Kruskal-Wallis H	1.446	4.798	9.458	3.764	2.071	8.998	12.791	17.157
df	2	2	2	2	2	2	2	2
Asymp. Sig.	.485	.091	.009	.152	.355	.011	.002	.000

a. Kruskal Wallis Test b. Grouping Variable: CHB, CHC, CHB+C

Table S11. Post-hoc Pairwise Comparisons

Ranks

	CHB, CHC, CHB+C	N	Mean Rank	Sum of Ranks
CD5L H-score	CHB	29	19.22	557.50
	CHC	15	28.83	432.50
	Total	44		

Test Statistics[a]

	CD5L H-score
Mann-Whitney U	122.500
Wilcoxon W	557.500
Z	-2.358
Asymp. Sig. (2-tailed)	.018

a. Grouping Variable: CHB, CHC, CHB+C

Ranks

	CHB, CHC, CHB+C	N	Mean Rank	Sum of Ranks
CD5L H-score	CHB	29	17.28	501.00
	CHB+C	10	27.90	279.00
	Total	39		

Test Statistics[a]

	CD5L H-score
Mann-Whitney U	66.000
Wilcoxon W	501.000
Z	-2.548
Asymp. Sig. (2-tailed)	.011
Exact Sig. [2*(1-tailed Sig.)]	.010[b]

a. Grouping Variable: CHB, CHC, CHB+C b. Not corrected for ties.

Ranks

	CHB, CHC, CHB+C	N	Mean Rank	Sum of Ranks
CD5L H-score	CHC	15	12.20	183.00
	CHB+C	10	14.20	142.00
	Total	25		

Test Statistics[a]

	CD5L H-score
Mann-Whitney U	63.000
Wilcoxon W	183.000
Z	-.669
Asymp. Sig. (2-tailed)	.503
Exact Sig. [2*(1-tailed Sig.)]	.531[b]

a. Grouping Variable: CHB, CHC, CHB+C. b. Not corrected for ties.

Ranks

	CHB, CHC, CHB+C	N	Mean Rank	Sum of Ranks
AST(U/L)	CHB	29	20.83	604.00
	CHC	15	25.73	386.00
	Total	44		

Test Statistics[a]

	AST(U/L)
Mann-Whitney U	169.000
Wilcoxon W	604.000
Z	-1.204
Asymp. Sig. (2-tailed)	.229

a. Grouping Variable: CHB, CHC, CHB+C

Ranks

	CHB, CHC, CHB+C	N	Mean Rank	Sum of Ranks
AST(U/L)	CHB	29	16.98	492.50
	CHB+C	10	28.75	287.50
	Total	39		

Test Statistics[a]

	AST(U/L)
Mann-Whitney U	57.500
Wilcoxon W	492.500
Z	-2.819
Asymp. Sig. (2-tailed)	.005

Exact Sig. [2*(1-tailed Sig.)]	.004[b]

a. Grouping Variable: CHB, CHC, CHB+C b. Not corrected for ties.

Ranks

	CHB, CHC, CHB+C	N	Mean Rank	Sum of Ranks
AST(U/L)	CHC	15	10.47	157.00
	CHB+C	10	16.80	168.00
	Total	25		

Test Statistics[a]

	AST(U/L)
Mann-Whitney U	37.000
Wilcoxon W	157.000
Z	-2.114
Asymp. Sig. (2-tailed)	.035
Exact Sig. [2*(1-tailed Sig.)]	.036[b]

a. Grouping Variable: CHB, CHC, CHB+C. b. Not corrected for ties.

Ranks

	CHB, CHC, CHB+C	N	Mean Rank	Sum of Ranks
ALT (U/L)	CHB	29	24.34	706.00
	CHC	15	18.93	284.00
	Total	44		

Test Statistics[a]

	ALT (U/L)
Mann-Whitney U	164.000
Wilcoxon W	284.000
Z	-1.327
Asymp. Sig. (2-tailed)	.184

a. Grouping Variable: CHB, CHC, CHB+C

Ranks

	CHB, CHC, CHB+C	N	Mean Rank	Sum of Ranks
ALT (U/L)	CHB	29	16.84	488.50
	CHB+C	10	29.15	291.50
	Total	39		

Test Statistics[a]

	ALT (U/L)

Mann-Whitney U	53.500
Wilcoxon W	488.500
Z	-2.945
Asymp. Sig. (2-tailed)	.003
Exact Sig. [2*(1-tailed Sig.)]	.002[b]

a. Grouping Variable: CHB, CHC, CHB+C, b. Not corrected for ties.

Ranks

	CHB, CHC, CHB+C	N	Mean Rank	Sum of Ranks
ALT (U/L)	CHC	15	9.07	136.00
	CHB+C	10	18.90	189.00
	Total	25		

Test Statistics[a]

	ALT (U/L)
Mann-Whitney U	16.000
Wilcoxon W	136.000
Z	-3.282
Asymp. Sig. (2-tailed)	.001
Exact Sig. [2*(1-tailed Sig.)]	.001[b]

a. Grouping Variable: CHB, CHC, CHB+C. b. Not corrected for ties.

Ranks

	CHB, CHC, CHB+C	N	Mean Rank	Sum of Ranks
GGT (U/L)	CHB	29	18.67	541.50
	CHC	15	29.90	448.50
	Total	44		

Test Statistics[a]

	GGT (U/L)
Mann-Whitney U	106.500
Wilcoxon W	541.500
Z	-2.754
Asymp. Sig. (2-tailed)	.006

a. Grouping Variable: CHB, CHC, CHB+C

Ranks

	CHB, CHC, CHB+C	N	Mean Rank	Sum of Ranks
GGT (U/L)	CHB	29	16.02	464.50
	CHB+C	10	31.55	315.50
	Total	39		

Test Statistics[a]

	GGT (U/L)
Mann-Whitney U	29.500
Wilcoxon W	464.500
Z	-3.721
Asymp. Sig. (2-tailed)	.000
Exact Sig. [2*(1-tailed Sig.)]	.000[b]

a. Grouping Variable: CHB, CHC, CHB+C. b. Not corrected for ties.

Ranks

	CHB, CHC, CHB+C	N	Mean Rank	Sum of Ranks
GGT (U/L)	CHC	15	11.33	170.00
	CHB+C	10	15.50	155.00
	Total	25		

Test Statistics[a]

	GGT (U/L)
Mann-Whitney U	50.000
Wilcoxon W	170.000
Z	-1.389
Asymp. Sig. (2-tailed)	.165
Exact Sig. [2*(1-tailed Sig.)]	.177[b]

a. Grouping Variable: CHB, CHC, CHB+C. b. Not corrected for ties.

Table S12. Distribution of MELD 3.0 across Child–Pugh classes

Case Processing Summary

		Cases					
		Valid		Missing		Total	
	CHILD PUGH	N	Percent	N	Percent	N	Percent
MELD 3.0	A	3	100.0%	0	0.0%	3	100.0%
	B	29	100.0%	0	0.0%	29	100.0%
	C	22	100.0%	0	0.0%	22	100.0%
MELD	A	3	100.0%	0	0.0%	3	100.0%
	B	29	100.0%	0	0.0%	29	100.0%
	C	22	100.0%	0	0.0%	22	100.0%

Descriptives

	CHILD PUGH			Statistic	Std. Error
MELD 3.0	A	Mean		16.6667	.33333
		95% Confidence Interval for Mean	Lower Bound	15.2324	
			Upper Bound	18.1009	
		5% Trimmed Mean		.	
		Median		17.0000	
		Variance		.333	
		Std. Deviation		.57735	
		Minimum		16.00	
		Maximum		17.00	
		Range		1.00	
		Interquartile Range		.	
		Skewness		-1.732	1.225
		Kurtosis		.	.
	B	Mean		18.1379	.20296
		95% Confidence Interval for Mean	Lower Bound	17.7222	
			Upper Bound	18.5537	
		5% Trimmed Mean		18.0977	
		Median		18.0000	
		Variance		1.195	
		Std. Deviation		1.09297	
		Minimum		16.00	
		Maximum		21.00	
		Range		5.00	
		Interquartile Range		1.00	
		Skewness		.766	.434
		Kurtosis		.861	.845
	C	Mean		20.1818	.29889
		95% Confidence Interval for Mean	Lower Bound	19.5602	
			Upper Bound	20.8034	
		5% Trimmed Mean		20.0404	
		Median		20.0000	

		Variance		1.965	
		Std. Deviation		1.40192	
		Minimum		19.00	
		Maximum		24.00	
		Range		5.00	
		Interquartile Range		2.00	
		Skewness		1.357	.491
		Kurtosis		1.540	.953
MELD	A	Mean		7.3333	.33333
		95% Confidence Interval for Mean	Lower Bound	5.8991	
			Upper Bound	8.7676	
		5% Trimmed Mean		.	
		Median		7.0000	
		Variance		.333	
		Std. Deviation		.57735	
		Minimum		7.00	
		Maximum		8.00	
		Range		1.00	
		Interquartile Range		.	
		Skewness		1.732	1.225
		Kurtosis		.	.
	B	Mean		7.6552	.27755
		95% Confidence Interval for Mean	Lower Bound	7.0866	
			Upper Bound	8.2237	
		5% Trimmed Mean		7.5613	
		Median		7.0000	
		Variance		2.234	
		Std. Deviation		1.49465	
		Minimum		6.00	
		Maximum		11.00	
		Range		5.00	
		Interquartile Range		2.00	
		Skewness		.918	.434

		Kurtosis		-.059	.845
	C	Mean		7.8182	.43463
		95% Confidence Interval for Mean	Lower Bound	6.9143	
			Upper Bound	8.7220	
		5% Trimmed Mean		7.5859	
		Median		7.0000	
		Variance		4.156	
		Std. Deviation		2.03859	
		Minimum		6.00	
		Maximum		14.00	
		Range		8.00	
		Interquartile Range		2.25	
		Skewness		1.754	.491
		Kurtosis		3.351	.953

Tests of Normality

	CHILD PUGH	Kolmogorov-Smirnov[a] Statistic	df	Sig.	Shapiro-Wilk Statistic	df	Sig.
MELD 3.0	A	.385	3	.	.750	3	.000
	B	.309	29	.000	.872	29	.002
	C	.233	22	.003	.808	22	.001
MELD	A	.385	3	.	.750	3	.000
	B	.290	29	.000	.855	29	.001
	C	.237	22	.002	.800	22	.001

a. Lilliefors Significance Correction

Table S13. Frequencies of histological features

Occasional isolation nodules

		Frequency	Percent	Valid Percent	Cumulative Percent
Valid	no	35	60.3	64.8	64.8
	yes	19	32.8	35.2	100.0
	Total	54	93.1	100.0	
Missing	System	4	6.9		

Total		58	100.0		

Cirrhosis

		Frequency	Percent	Valid Percent	Cumulative Percent
Valid	no	36	62.1	66.7	66.7
	yes	18	31.0	33.3	100.0
	Total	54	93.1	100.0	
Missing	System	4	6.9		
Total		58	100.0		

Table S14. Comparative histological features across CHB, CHC, and CHB+C groups

		Portal inflammatory infiltrate	Lobular inflammatory infiltrate	Interface hepatitis	Isolated intralobular hepatocyte necrosis	Intralobular confluent hepatocyte necrosis	Hepatocyte apoptosis	Dystrophic lesions	Ballooning degeneration	Steatosis	Intrahepatocyte pigment	Ground glass hepatocytes	Others	Kupffer cells hypertrophy	Kupffer cells hyperplasia	Intrasinusoidal lymphocyte infiltration	Sinusoidal dilation	Biliary duct lesions	Portal fibrosis	Septal fibrosis	Fibrous bridges	Occasional isolation nodules	Cirrhosis
N	Valid	54	54	54	54	54	54	54	54	54	54	54	54	54	54	54	54	54	54	54	54	54	54
	Missing	4	4	4	4	4	4	4	4	4	4	4	4	4	4	4	4	4	4	4	4	4	4
Percentiles	25	1.0000	2.0000	2.0000	1.0000	.0000	1.0000	1.0000	1.0000	.0000	.0000	.0000	.0000	1.0000	1.0000	.0000	.0000	1.0000	1.0000	1.0000	.0000	.0000	.0000
	50	1.0000	2.0000	2.0000	1.0000	.0000	1.0000	1.0000	1.0000	1.0000	.0000	1.0000	.0000	1.0000	1.0000	.0000	.0000	1.0000	1.0000	1.0000	1.0000	.0000	.0000
	75	1.0000	2.0000	2.0000	2.0000	.0000	2.0000	1.0000	1.0000	2.0000	.0000	1.0000	1.0000	1.0000	1.0000	.0000	.0000	2.0000	1.0000	1.0000	1.0000	1.0000	1.0000

Portal inflammatory infiltrate

		Frequency	Percent	Valid Percent	Cumulative Percent

Valid	moderate	48	82.8	88.9	88.9
	minimum	6	10.3	11.1	100.0
	Total	54	93.1	100.0	
Missing	System	4	6.9		
Total		58	100.0		

Lobular inflammatory infiltrate

		Frequency	Percent	Valid Percent	Cumulative Percent
Valid	moderate	5	8.6	9.3	9.3
	minimum	49	84.5	90.7	100.0
	Total	54	93.1	100.0	
Missing	System	4	6.9		
Total		58	100.0		

Interface hepatitis

		Frequency	Percent	Valid Percent	Cumulative Percent
Valid	minimum	11	19.0	20.4	20.4
	moderate, continuous	35	60.3	64.8	85.2
	marked, continuous	8	13.8	14.8	100.0
	Total	54	93.1	100.0	
Missing	System	4	6.9		
Total		58	100.0		

Isolated intralobular hepatocyte necrosis

		Frequency	Percent	Valid Percent	Cumulative Percent
Valid	rare	36	62.1	66.7	66.7
	multiple	18	31.0	33.3	100.0
	Total	54	93.1	100.0	
Missing	System	4	6.9		
Total		58	100.0		

Intralobular confluent hepatocyte necrosis

		Frequency	Percent	Valid Percent	Cumulative Percent
Valid	no	54	93.1	100.0	100.0
Missing	System	4	6.9		
Total		58	100.0		

Hepatocyte apoptosis

		Frequency	Percent	Valid Percent	Cumulative Percent
Valid	Condensed hepatocites +Councilman	30	51.7	55.6	55.6
	Condensed hepatocites +Councilman	24	41.4	44.4	100.0
	Total	54	93.1	100.0	
Missing	System	4	6.9		
Total		58	100.0		

Dystrophic lesions

		Frequency	Percent	Valid Percent	Cumulative Percent
Valid	Yes	54	93.1	100.0	100.0
Missing	System	4	6.9		
Total		58	100.0		

Ballooning degeneration

		Frequency	Percent	Valid Percent	Cumulative Percent
Valid	present	50	86.2	92.6	92.6
	important	4	6.9	7.4	100.0
	Total	54	93.1	100.0	
Missing	System	4	6.9		
Total		58	100.0		

Steatosis

		Frequency	Percent	Valid Percent	Cumulative Percent
Valid	no	14	24.1	25.9	25.9
	minimum	21	36.2	38.9	64.8
	ocassional	19	32.8	35.2	100.0
	Total	54	93.1	100.0	
Missing	System	4	6.9		
Total		58	100.0		

Intrahepatocyte pigment

		Frequency	Percent	Valid Percent	Cumulative Percent
Valid	no	54	93.1	100.0	100.0
Missing	System	4	6.9		
Total		58	100.0		

Ground glass hepatocytes

		Frequency	Percent	Valid Percent	Cumulative Percent
Valid	no	23	39.7	42.6	42.6
	yes	20	34.5	37.0	79.6
	numerous	11	19.0	20.4	100.0
	Total	54	93.1	100.0	
Missing	System	4	6.9		
Total		58	100.0		

Others

		Frequency	Percent	Valid Percent	Cumulative Percent
Valid	no	32	55.2	59.3	59.3
	glycogen nuclei	10	17.2	18.5	77.8
	rosettes	11	19.0	20.4	98.1
	mitotic hepatocytes	1	1.7	1.9	100.0
	Total	54	93.1	100.0	
Missing	System	4	6.9		
Total		58	100.0		

Kupffer cells hypertrophy

		Frequency	Percent	Valid Percent	Cumulative Percent
Valid	minimal, focal	53	91.4	98.1	98.1
	moderate, focal	1	1.7	1.9	100.0
	Total	54	93.1	100.0	
Missing	System	4	6.9		
Total		58	100.0		

Kupffer cells hyperplasia

		Frequency	Percent	Valid Percent	Cumulative Percent
Valid	minimal, focal	53	91.4	98.1	98.1
	moderate, focal	1	1.7	1.9	100.0
	Total	54	93.1	100.0	
Missing	System	4	6.9		
Total		58	100.0		

Intrasinusoidal lymphocyte infiltration

		Frequency	Percent	Valid Percent	Cumulative Percent
Valid	no	54	93.1	100.0	100.0
Missing	System	4	6.9		
Total		58	100.0		

Sinusoidal dilation

		Frequency	Percent	Valid Percent	Cumulative Percent
Valid	no	54	93.1	100.0	100.0
Missing	System	4	6.9		
Total		58	100.0		

Biliary duct lesions

		Frequency	Percent	Valid Percent	Cumulative Percent
Valid	no	11	19.0	20.4	20.4
	yes	29	50.0	53.7	74.1
	neocanalicular proliferation	14	24.1	25.9	100.0
	Total	54	93.1	100.0	
Missing	System	4	6.9		
Total		58	100.0		

Portal fibrosis

		Frequency	Percent	Valid Percent	Cumulative Percent
Valid	moderate	42	72.4	77.8	77.8
	advanced	12	20.7	22.2	100.0
	Total	54	93.1	100.0	
Missing	System	4	6.9		
Total		58	100.0		

Septal fibrosis

		Frequency	Percent	Valid Percent	Cumulative Percent
Valid	no	8	13.8	14.8	14.8
	yes	46	79.3	85.2	100.0
	Total	54	93.1	100.0	

Missing	System	4	6.9		
Total		58	100.0		

Fibrous bridges

		Frequency	Percent	Valid Percent	Cumulative Percent
Valid	no	18	31.0	33.3	33.3
	yes	36	62.1	66.7	100.0
	Total	54	93.1	100.0	
Missing	System	4	6.9		
Total		58	100.0		

Descriptive Statistics

	N	Percentiles 25th	50th (Median)	75th
Portal inflammatory infiltrate	54	1.0000	1.0000	1.0000
Lobular inflammatory infiltrate	54	2.0000	2.0000	2.0000
Interface hepatitis	54	2.0000	2.0000	2.0000
Isolated intralobular hepatocyte necrosis	54	1.0000	1.0000	2.0000
Intralobular confluent hepatocyte necrosis	54	.0000	.0000	.0000
Hepatocyte apoptosis	54	1.0000	1.0000	2.0000
Dystrophic lesions	54	1.0000	1.0000	1.0000
Ballooning degeneration	54	1.0000	1.0000	1.0000
Steatosis	54	.0000	1.0000	2.0000
Intrahepatocyte pigment	54	.0000	.0000	.0000
Ground glass hepatocytes	54	.0000	1.0000	1.0000
Others	54	.0000	.0000	1.0000
Kupffer cells hypertrophy	54	1.0000	1.0000	1.0000
Kupffer cells hyperplasia	54	1.0000	1.0000	1.0000
Intrasinusoidal lymphocyte infiltration	54	.0000	.0000	.0000
Sinusoidal dilation	54	.0000	.0000	.0000
Biliary duct lesions	54	1.0000	1.0000	2.0000
Portal fibrosis	54	1.0000	1.0000	1.0000

Septal fibrosis	54	1.0000	1.0000	1.0000
Fibrous bridges	54	.0000	1.0000	1.0000
Occasional isolation nodules	54	.0000	.0000	1.0000
Cirrhosis	54	.0000	.0000	1.0000
CHB, CHC, CHB+C	54	1.0000	1.0000	2.0000

Ranks

	CHB, CHC, CHB+C	N	Mean Rank
Portal inflammatory infiltrate	CHB	29	30.09
	CHC	15	24.50
	CHB+C	10	24.50
	Total	54	
Lobular inflammatory infiltrate	CHB	29	30.00
	CHC	15	30.00
	CHB+C	10	16.50
	Total	54	
Interface hepatitis	CHB	29	23.24
	CHC	15	29.00
	CHB+C	10	37.60
	Total	54	
Isolated intralobular hepatocyte necrosis	CHB	29	32.47
	CHC	15	18.50
	CHB+C	10	26.60
	Total	54	
Intralobular confluent hepatocyte necrosis	CHB	29	27.50
	CHC	15	27.50
	CHB+C	10	27.50
	Total	54	
Hepatocyte apoptosis	CHB	29	29.47
	CHC	15	26.30
	CHB+C	10	23.60
	Total	54	
Dystrophic lesions	CHB	29	27.50
	CHC	15	27.50
	CHB+C	10	27.50
	Total	54	
Ballooning degeneration	CHB	29	25.50
	CHC	15	27.30
	CHB+C	10	33.60

	Total	54	
Steatosis	CHB	29	26.55
	CHC	15	34.50
	CHB+C	10	19.75
	Total	54	
Intrahepatocyte pigment	CHB	29	27.50
	CHC	15	27.50
	CHB+C	10	27.50
	Total	54	
Ground glass hepatocytes	CHB	29	34.93
	CHC	15	12.00
	CHB+C	10	29.20
	Total	54	
Others	CHB	29	31.34
	CHC	15	20.70
	CHB+C	10	26.55
	Total	54	
Kupffer cells hypertrophy	CHB	29	27.00
	CHC	15	27.00
	CHB+C	10	29.70
	Total	54	
Kupffer cells hyperplasia	CHB	29	27.00
	CHC	15	27.00
	CHB+C	10	29.70
	Total	54	
Intrasinusoidal lymphocyte infiltration	CHB	29	27.50
	CHC	15	27.50
	CHB+C	10	27.50
	Total	54	
Sinusoidal dilation	CHB	29	27.50
	CHC	15	27.50
	CHB+C	10	27.50
	Total	54	
Biliary duct lesions	CHB	29	25.03
	CHC	15	30.40
	CHB+C	10	30.30
	Total	54	
Portal fibrosis	CHB	29	25.22

	CHC	15	34.10
	CHB+C	10	24.20
	Total	54	
Septal fibrosis	CHB	29	24.05
	CHC	15	31.50
	CHB+C	10	31.50
	Total	54	
Fibrous bridges	CHB	29	23.47
	CHC	15	32.90
	CHB+C	10	31.10
	Total	54	
Occasional isolation nodules	CHB	29	26.38
	CHC	15	30.60
	CHB+C	10	26.10
	Total	54	
Cirrhosis	CHB	29	25.95
	CHC	15	31.10
	CHB+C	10	26.60
	Total	54	

Frequencies

		CHB, CHC, CHB+C		
		CHB	CHC	CHB+C
Portal inflammatory infiltrate	> Median	6	0	0
	<= Median	23	15	10
Lobular inflammatory infiltrate	> Median	0	0	0
	<= Median	29	15	10
Interface hepatitis	> Median	4	0	4
	<= Median	25	15	6
Isolated intralobular hepatocyte necrosis	> Median	15	0	3
	<= Median	14	15	7
Intralobular confluent hepatocyte necrosis	> Median	0	0	0
	<= Median	29	15	10
Hepatocyte apoptosis	> Median	15	6	3
	<= Median	14	9	7
Dystrophic lesions	> Median	0	0	0
	<= Median	29	15	10
Ballooning degeneration	> Median	0	1	3
	<= Median	29	14	7

Steatosis	> Median	11	8	0
	<= Median	18	7	10
Intrahepatocyte pigment	> Median	0	0	0
	<= Median	29	15	10
Ground glass hepatocytes	> Median	11	0	0
	<= Median	18	15	10
Others	> Median	15	3	4
	<= Median	14	12	6
Kupffer cells hypertrophy	> Median	0	0	1
	<= Median	29	15	9
Kupffer cells hyperplasia	> Median	0	0	1
	<= Median	29	15	9
Intrasinusoidal lymphocyte infiltration	> Median	0	0	0
	<= Median	29	15	10
Sinusoidal dilation	> Median	0	0	0
	<= Median	29	15	10
Biliary duct lesions	> Median	8	4	2
	<= Median	21	11	8
Portal fibrosis	> Median	4	7	1
	<= Median	25	8	9
Septal fibrosis	> Median	0	0	0
	<= Median	29	15	10
Fibrous bridges	> Median	0	0	0
	<= Median	29	15	10
Occasional isolation nodules	> Median	9	7	3
	<= Median	20	8	7
Cirrhosis	> Median	8	7	3
	<= Median	21	8	7

Ranks

	CHB, CHC, CHB+C	N	Mean Rank	Sum of Ranks
Portal inflammatory infiltrate	CHB	29	24.05	697.50
	CHC	15	19.50	292.50
	Total	44		
Lobular inflammatory infiltrate	CHB	29	22.50	652.50
	CHC	15	22.50	337.50
	Total	44		
Interface hepatitis	CHB	29	20.69	600.00
	CHC	15	26.00	390.00
	Total	44		

Isolated intralobular hepatocyte necrosis	CHB	29	26.38	765.00
	CHC	15	15.00	225.00
	Total	44		
Intralobular confluent hepatocyte necrosis	CHB	29	22.50	652.50
	CHC	15	22.50	337.50
	Total	44		
Hepatocyte apoptosis	CHB	29	23.38	678.00
	CHC	15	20.80	312.00
	Total	44		
Dystrophic lesions	CHB	29	22.50	652.50
	CHC	15	22.50	337.50
	Total	44		
Ballooning degeneration	CHB	29	22.00	638.00
	CHC	15	23.47	352.00
	Total	44		
Steatosis	CHB	29	20.45	593.00
	CHC	15	26.47	397.00
	Total	44		
Intrahepatocyte pigment	CHB	29	22.50	652.50
	CHC	15	22.50	337.50
	Total	44		
Ground glass hepatocytes	CHB	29	28.45	825.00
	CHC	15	11.00	165.00
	Total	44		
Others	CHB	29	25.45	738.00
	CHC	15	16.80	252.00
	Total	44		
Kupffer cells hypertrophy	CHB	29	22.50	652.50
	CHC	15	22.50	337.50
	Total	44		
Kupffer cells hyperplasia	CHB	29	22.50	652.50
	CHC	15	22.50	337.50
	Total	44		
Intrasinusoidal lymphocyte infiltration	CHB	29	22.50	652.50
	CHC	15	22.50	337.50
	Total	44		
Sinusoidal dilation	CHB	29	22.50	652.50

	CHC	15	22.50	337.50
	Total	44		
Biliary duct lesions	CHB	29	21.03	610.00
	CHC	15	25.33	380.00
	Total	44		
Portal fibrosis	CHB	29	20.03	581.00
	CHC	15	27.27	409.00
	Total	44		
Septal fibrosis	CHB	29	20.43	592.50
	CHC	15	26.50	397.50
	Total	44		
Fibrous bridges	CHB	29	19.88	576.50
	CHC	15	27.57	413.50
	Total	44		
Occasional isolation nodules	CHB	29	21.33	618.50
	CHC	15	24.77	371.50
	Total	44		
Cirrhosis	CHB	29	21.07	611.00
	CHC	15	25.27	379.00
	Total	44		

Test Statistics[a,d,g,i,l,p,q,t,u]

	Portal inflammatory infiltrate	Lobular inflammatory infiltrate	Interface hepatitis	Isolated intralobular hepatocyte necrosis	Intralobular confluent hepatocyte necrosis	Hepatocyte apoptosis	Dystrophic lesions	Ballooning degeneration	Steatosis	Intrahepatocyte pigment	Ground glass hepatocytes
N	54	54	54	54	54	54	54	54	54	54	54
Median	1.0000	2.0000[c]	2.0000	1.0000	.0000[c]	1.0000	1.0000[c]	1.0000	1.0000	.0000[c]	1.0000
Chi-Square	5.819[b]		7.659[e]	11.964[f]		1.587[h]		9.774[j]	7.691[k]		11.909[m]
df	2		2	2		2		2	2		2
Asymp. Sig.	.055		.022	.003		.452		.008	.021		.003

	Others	Kupffer cells hypertrophy	Kupffer cells hyperplasia	Intrasinusoidal lymphocyte infiltration	Sinusoidal dilation	Biliary duct lesions	Portal fibrosis	Septal fibrosis	Fibrous bridges	Occasional isolation nodules	Cirrhosis
N	54	54	54	54	54	54	54	54	54	54	54
Median	.0000	1.0000	1.0000	.0000[c]	.0000[c]	1.0000	1.0000	1.0000[c]	1.0000[c]	.0000	.0000
Chi-Square	4.124[n]	4.483[o]	4.483[o]			.229[r]	7.242[s]			1.204[k]	1.681[f]
df	2	2	2			2	2			2	2
Asymp. Sig.	.127	.106	.106			.892	.027			.548	.431

a. Grouping Variable: CHB, CHC, CHB+C

b. 3 cells (50.0%) have expected frequencies less than 5. The minimum expected cell frequency is 1.1.

c. All values are less than or equal to the median. Median Test cannot be performed.

d. There are not enough valid cases to perform the Median Test for Lobular inflammatory infiltrate * CHB, CHC, CHB+C (CHB, CHB+C). No statistics are computed.

e. 3 cells (50.0%) have expected frequencies less than 5. The minimum expected cell frequency is 1.5.

f. 1 cells (16.7%) have expected frequencies less than 5. The minimum expected cell frequency is 3.3.

g. There are not enough valid cases to perform the Median Test for Intralobular confluent hepatocyte necrosis * CHB, CHC, CHB+C (CHB, CHB+C). No statistics are computed.

h. 1 cells (16.7%) have expected frequencies less than 5. The minimum expected cell frequency is 4.4.

i. There are not enough valid cases to perform the Median Test for Dystrophic lesions * CHB, CHC, CHB+C (CHB, CHB+C). No statistics are computed.

j. 3 cells (50.0%) have expected frequencies less than 5. The minimum expected cell frequency is .7.

k. 1 cells (16.7%) have expected frequencies less than 5. The minimum expected cell frequency is 3.5.

l. There are not enough valid cases to perform the Median Test for Intrahepatocyte pigment * CHB, CHC, CHB+C (CHB, CHB+C). No statistics are computed.

m. 2 cells (33.3%) have expected frequencies less than 5. The minimum expected cell frequency is 2.0.

n. 1 cells (16.7%) have expected frequencies less than 5. The minimum expected cell frequency is 4.1.

o. 3 cells (50.0%) have expected frequencies less than 5. The minimum expected cell frequency is .2.

p. There are not enough valid cases to perform the Median Test for Intrasinusoidal lymphocyte infiltration * CHB, CHC, CHB+C (CHB, CHB+C). No statistics are computed.

q. There are not enough valid cases to perform the Median Test for Sinusoidal dilation * CHB, CHC, CHB+C (CHB, CHB+C). No statistics are computed.

r. 2 cells (33.3%) have expected frequencies less than 5. The minimum expected cell frequency is 2.6.

s. 2 cells (33.3%) have expected frequencies less than 5. The minimum expected cell frequency is 2.2.

t. There are not enough valid cases to perform the Median Test for Septal fibrosis * CHB, CHC, CHB+C (CHB, CHB+C). No statistics are computed.

u. There are not enough valid cases to perform the Median Test for Fibrous bridges * CHB, CHC, CHB+C (CHB, CHB+C). No statistics are computed.

Ranks

	CHB, CHC, CHB+C	N	Mean Rank	Sum of Ranks
Portal inflammatory infiltrate	CHB	29	21.03	610.00
	CHB+C	10	17.00	170.00
	Total	39		
Lobular inflammatory infiltrate	CHB	29	22.50	652.50
	CHB+C	10	12.75	127.50
	Total	39		
Interface hepatitis	CHB	29	17.55	509.00
	CHB+C	10	27.10	271.00
	Total	39		
Isolated intralobular hepatocyte necrosis	CHB	29	21.09	611.50
	CHB+C	10	16.85	168.50
	Total	39		
Intralobular confluent hepatocyte necrosis	CHB	29	20.00	580.00
	CHB+C	10	20.00	200.00
	Total	39		
Hepatocyte apoptosis	CHB	29	21.09	611.50
	CHB+C	10	16.85	168.50

	Total	39		
Dystrophic lesions	CHB	29	20.00	580.00
	CHB+C	10	20.00	200.00
	Total	39		
Ballooning degeneration	CHB	29	18.50	536.50
	CHB+C	10	24.35	243.50
	Total	39		
Steatosis	CHB	29	21.10	612.00
	CHB+C	10	16.80	168.00
	Total	39		
Intrahepatocyte pigment	CHB	29	20.00	580.00
	CHB+C	10	20.00	200.00
	Total	39		
Ground glass hepatocytes	CHB	29	21.48	623.00
	CHB+C	10	15.70	157.00
	Total	39		
Others	CHB	29	20.90	606.00
	CHB+C	10	17.40	174.00
	Total	39		
Kupffer cells hypertrophy	CHB	29	19.50	565.50
	CHB+C	10	21.45	214.50
	Total	39		
Kupffer cells hyperplasia	CHB	29	19.50	565.50
	CHB+C	10	21.45	214.50
	Total	39		
Intrasinusoidal lymphocyte infiltration	CHB	29	20.00	580.00
	CHB+C	10	20.00	200.00
	Total	39		
Sinusoidal dilation	CHB	29	20.00	580.00
	CHB+C	10	20.00	200.00
	Total	39		
Biliary duct lesions	CHB	29	19.00	551.00
	CHB+C	10	22.90	229.00
	Total	39		
Portal fibrosis	CHB	29	20.19	585.50
	CHB+C	10	19.45	194.50
	Total	39		

		N	Mean Rank	Sum of Ranks
Septal fibrosis	CHB	29	18.62	540.00
	CHB+C	10	24.00	240.00
	Total	39		
Fibrous bridges	CHB	29	18.59	539.00
	CHB+C	10	24.10	241.00
	Total	39		
Occasional isolation nodules	CHB	29	20.05	581.50
	CHB+C	10	19.85	198.50
	Total	39		
Cirrhosis	CHB	29	19.88	576.50
	CHB+C	10	20.35	203.50
	Total	39		

Test Statistics[a]

	Portal inflammatory infiltrate	Lobular inflammatory infiltrate	Interface hepatitis	Isolated intralobular hepatocyte necrosis	Intralobular confluent hepatocyte necrosis	Hepatocyte apoptosis	Dystrophic lesions	Ballooning degeneration	Steatosis	Intrahepatocyte pigment	Ground glass hepatocytes
Mann-Whitney U	115.000	72.500	74.000	113.500	145.000	113.500	145.000	101.500	113.000	145.000	102.000
Wilcoxon W	170.000	127.500	509.000	168.500	200.000	168.500	200.000	536.500	168.000	200.000	157.000
Z	-1.544	-4.026	-2.500	-1.173	.000	-1.173	.000	-3.030	-1.095	.000	-1.514
Asymp. Sig. (2-tailed)	.123	<.001	.012	.241	1.000	.241	1.000	.002	.274	1.000	.130
Exact Sig. [2*(1-tailed Sig.)]	.348[b]	.018[b]	.022[b]	.316[b]	1.000[b]	.316[b]	1.000[b]	.164[b]	.316[b]	1.000[b]	.174[b]

	Others	Kupffer cells hypertrophy	Kupffer cells hyperplasia	Intrasinusoidal lymphocyte infiltration	Sinusoidal dilation	Biliary duct lesions	Portal fibrosis	Septal fibrosis	Fibrous bridges	Occasional isolation nodules	Cirrhosis
Mann-Whitney U	119.000	130.500	130.500	145.000	145.000	116.000	139.500	105.000	104.000	143.500	141.500
Wilcoxon W	174.000	565.500	565.500	200.000	200.000	551.000	194.500	540.000	539.000	198.500	576.500
Z	-.914	-1.703	-1.703	.000	.000	-1.011	-.305	-1.839	-1.547	-.060	-.144
Asymp. Sig. (2-tailed)	.361	.089	.089	1.000	1.000	.312	.760	.066	.122	.952	.885
Exact Sig. [2*(1-tailed Sig.)]	.418[b]	.646[b]	.646[b]	1.000[b]	1.000[b]	.365[b]	.862[b]	.208[b]	.196[b]	.962[b]	.912[b]

a. Grouping Variable: CHB, CHC, CHB+C

b. Not corrected for ties.

Ranks

	CHB, CHC, CHB+C	N	Mean Rank	Sum of Ranks
Portal inflammatory infiltrate	CHC	15	13.00	195.00
	CHB+C	10	13.00	130.00
	Total	25		
Lobular inflammatory infiltrate	CHC	15	15.50	232.50
	CHB+C	10	9.25	92.50
	Total	25		
Interface hepatitis	CHC	15	11.00	165.00
	CHB+C	10	16.00	160.00
	Total	25		
Isolated intralobular hepatocyte necrosis	CHC	15	11.50	172.50
	CHB+C	10	15.25	152.50
	Total	25		
Intralobular confluent hepatocyte necrosis	CHC	15	13.00	195.00
	CHB+C	10	13.00	130.00
	Total	25		
Hepatocyte apoptosis	CHC	15	13.50	202.50

	CHB+C	10	12.25	122.50
	Total	25		
Dystrophic lesions	CHC	15	13.00	195.00
	CHB+C	10	13.00	130.00
	Total	25		
Ballooning degeneration	CHC	15	11.83	177.50
	CHB+C	10	14.75	147.50
	Total	25		
Steatosis	CHC	15	16.03	240.50
	CHB+C	10	8.45	84.50
	Total	25		
Intrahepatocyte pigment	CHC	15	13.00	195.00
	CHB+C	10	13.00	130.00
	Total	25		
Ground glass hepatocytes	CHC	15	9.00	135.00
	CHB+C	10	19.00	190.00
	Total	25		
Others	CHC	15	11.90	178.50
	CHB+C	10	14.65	146.50
	Total	25		
Kupffer cells hypertrophy	CHC	15	12.50	187.50
	CHB+C	10	13.75	137.50
	Total	25		
Kupffer cells hyperplasia	CHC	15	12.50	187.50
	CHB+C	10	13.75	137.50
	Total	25		
Intrasinusoidal lymphocyte infiltration	CHC	15	13.00	195.00
	CHB+C	10	13.00	130.00
	Total	25		
Sinusoidal dilation	CHC	15	13.00	195.00
	CHB+C	10	13.00	130.00
	Total	25		
Biliary duct lesions	CHC	15	13.07	196.00
	CHB+C	10	12.90	129.00
	Total	25		
Portal fibrosis	CHC	15	14.83	222.50
	CHB+C	10	10.25	102.50

	Total	25		
Septal fibrosis	CHC	15	13.00	195.00
	CHB+C	10	13.00	130.00
	Total	25		
Fibrous bridges	CHC	15	13.33	200.00
	CHB+C	10	12.50	125.00
	Total	25		
Occasional isolation nodules	CHC	15	13.83	207.50
	CHB+C	10	11.75	117.50
	Total	25		
Cirrhosis	CHC	15	13.83	207.50
	CHB+C	10	11.75	117.50
	Total	25		

Test Statistics[a]

	Portal inflammatory infiltrate	Lobular inflammatory infiltrate	Interface hepatitis	Isolated intralobular hepatocyte necrosis	Intralobular confluent hepatocyte necrosis	Hepatocyte apoptosis	Dystrophic lesions	Ballooning degeneration	Steatosis	Intrahepatocyte pigment	Ground glass hepatocytes	Others
Mann-Whitney U	75.000	37.500	45.000	52.500	75.000	67.500	75.000	57.500	29.500	75.000	15.000	58.500
Wilcoxon W	130.000	92.500	165.000	172.500	130.000	122.500	130.000	177.500	84.500	130.000	135.000	178.500
Z	.000	-3.000	-2.619	-2.216	.000	-.500	.000	-1.528	-2.781	.000	-4.116	-1.168
Asymp. Sig. (2-tailed)	1.000	.003	.009	.027	1.000	.617	1.000	.127	.005	1.000	<.001	.243
Exact Sig. [2*(1-tailed Sig.)]	1.000[b]	.036[b]	.103[b]	.216[b]	1.000[b]	.683[b]	1.000[b]	.338[b]	.010[b]	1.000[b]	<.001[b]	.367[b]

	Kupffer cells hypertrophy	Kupffer cells hyperplasia	Intrasinusoidal lymphocyte infiltration	Sinusoidal dilation	Biliary duct lesions	Portal fibrosis	Septal fibrosis	Fibrous bridges	Occasional isolation nodules	Cirrhosis
Mann-Whitney U	67.500	67.500	75.000	75.000	74.000	47.500	75.000	70.000	62.500	62.500
Wilcoxon W	187.500	187.500	130.000	130.000	129.000	102.500	130.000	125.000	117.500	117.500
Z	-1.225	-1.225	.000	.000	-.071	-1.886	.000	-.436	-.816	-.816
Asymp. Sig. (2-tailed)	.221	.221	1.000	1.000	.944	.059	1.000	.663	.414	.414
Exact Sig. [2*(1-tailed Sig.)]	.683[b]	.683[b]	1.000[b]	1.000[b]	.978[b]	.129[b]	1.000[b]	.807[b]	.495[b]	.495[b]

a. Grouping Variable: CHB, CHC, CHB+C

b. Not corrected for ties.
